# Comprehensive Analysis of 11 Species of *Euodia* (Rutaceae) by Untargeted LC-IT-TOF/MS Metabolomics and In Vitro Functional Methods

**DOI:** 10.3390/molecules29051059

**Published:** 2024-02-28

**Authors:** Xuhong Yong, Bi Wang, Mengdi Wang, Hui Lyu, Min Yin, Tong Jin, Xu Feng, Yu Shan, Yan Liang, Qizhi Wang

**Affiliations:** 1 Jiangsu Key Laboratory for the Research and Utilization of Plant Resources, Jiangsu Province Engineering Research Center of Eco-Cultivation and High-Value Utilization of Chinese Medicinal Materials, Institute of Botany, Jiangsu Province and Chinese Academy of Sciences, Nanjing 210014, China; 2 Nanjing University of Chinese Medicine, Nanjing 210023, China; 3 Key Lab of Drug Metabolism & Pharmacokinetics, State Key Laboratory of Natural Medicines, China Pharmaceutical University, Nanjing 210009, China

**Keywords:** untargeted metabolomics, *Euodia*, indole quinazoline alkaloids, bioactivities, resource exploitation

## Abstract

The *Euodia* genus comprises numerous untapped medicinal plants that warrant thorough evaluation for their potential as valuable natural sources of herbal medicine or food flavorings. In this study, untargeted metabolomics and in vitro functional methods were employed to analyze fruit extracts from 11 significant species of the *Euodia* genus. An investigation of the distribution of metabolites (quinolone and indole quinazoline alkaloids) in these species indicated that *E. rutaecarpa* (*Euodia rutaecarpa*) was the most widely distributed species, followed by *E. compacta* (*Euodia compacta*), *E. glabrifolia* (*Euodia glabrifolia*), *E. austrosinensis* (*Euodia austrosinensis*), and *E. fargesii* (*Euodia fargesii*). There have been reports on the close correlation between indole quinazoline alkaloids and their anti-tumor activity, especially in *E. rutaecarpa* fruits which exhibit effectiveness against various types of cancer, such as SGC-7901, Hela, A549, and other cancer cell lines. Additionally, the *E. rutaecarpa* plant contains indole quinazoline alkaloids, which possess remarkable antibacterial properties. Our results offer novel insights into the utilization of *Euodia* resources in the pharmaceutical industry.

## 1. Introduction

*Euodia* is one of the largest genera in the family Rutaceae, with approximately 150 species found in Asia, eastern Africa, and Oceania. In China, there are approximately 20 species and five varieties [1]. Due to its extensive use as a cooking seasoning in southwest China, and some species inclusion in the Pharmacopoeia of the People’s Republic of China 2020 Edition as a traditional Chinese medicine, it has important economic value. At present, a variety of secondary metabolites with potential therapeutic effects have been isolated in phytochemical studies of this genus, including indole quinazoline alkaloids, quinolone alkaloids, limonin compounds, and other components. These metabolites play an important role in the anti-tumor [2,3,4,5], antibacterial [6,7,8], anti-inflammatory [9,10], and insecticidal [11,12] properties of *Euodia*. Some secondary metabolites have already been applied in clinical treatments and show great potential as proprietary drugs. Evodiamine and rutaecarpine have shown significant efficacy in the prevention and treatment of Alzheimer’s disease [13], diabetes [14], and cardiovascular diseases [15]. In addition to the extensive research on the pharmacological effects of *Euodia*, dietary supplements containing this ingredient are also rapidly gaining popularity [16,17]. The pharmacological properties of *Euodia* have been widely utilized in the pharmaceutical industry. From an industrial perspective, the chemical composition and pharmacological properties of *E. rutaecarpa* (*Euodia rutaecarpa*) can be exploited in the pharmaceutical field, so the research and development of other species may help to drive new strategies for industrial profits from *Euodia*.

In recent years, the rapid development of metabolomics technology has made it one of the most promising analytical platforms in several fields. With the aim of systematically gathering the qualitative and quantitative data of low-molecular-mass endogenous metabolites, metabolomics is based primarily on the analytical technologies of nuclear magnetic resonance (NMR), gas chromatography (GC), and liquid chromatography (LC) coupled to mass spectrometry (MS) [18]. Metabolomics provides an analytical description of complex biological samples to characterize and quantify small-molecule metabolites [19]. With the support of MS-based untargeted metabolomics, it is possible to investigate a variety of natural plant products, research plant metabolism, and perform high-throughput metabolite screening [20]. Liquid chromatography–ion trap–time-of-flight/mass spectrometry (LC-IT-TOF/MS) can be used to assess numerous non-target metabolic characteristics, particularly for solving the problems of primary metabolism, complex secondary metabolites, and large plant systems. In fact, there have been many studies on the mechanisms underlying the pharmacological activities of *Euodia* alkaloids based on metabolomics technology [21,22,23,24]. In recent years, the field of metabolomics has gained significant traction as a valuable analytical tool for optimizing the potential health benefits of underutilized medicinal plants and associated substances. Tang et al. analyzed the total phenolic acid content, total flavonoid content and antioxidant capacity of 22 lily bulbs species by means of the metabolomics method. The results showed that the total phenolic acid content, total flavonoid content, and antioxidant activity of different lily species were significantly different, but showed a significant positive correlation [25]. Jiang et al. conducted a metabolomic study on the polyphenols contained in five varieties of litchi, and found significant differences among varieties, finding also that there was a certain correlation between the maturity of litchi varieties and the distribution and content of phenolic substances [26]. Liu et al. used GC-MS technology to analyze the differences in primary metabolites between honeysuckle and Lonicera cinerea, and the results showed that the significant differences in metabolites between honeysuckle and Lonicera cinerea were caused by the aggregation of galactose metabolism, starch metabolism, and sucrose metabolism as potential metabolic pathways [27]. This is primarily achieved through the implementation of untargeted approaches, enabling the comprehensive exploration of a diverse array of metabolites [28]. However, there continues to be a limited amount of comprehensive research on the variations in secondary metabolites present in *Euodia*, which hinders the progress of this genus’ industrial development and its potential utilization. In view of this, we hypothesize that the plants of the genus *Euodia* are rich in active ingredients and have broad prospects for development.

In this study, metabolites from 11 species of *Euodia* (*Euodia lepta (E. lepta*), *Euodia rutaecarpa* (*E. rutaecarpa*), *Euodia compacta* (*E. compacta*), *Euodia austrosinensis* (*E. austrosinensis*), *Euodia glabrifolia* (*E. glabrifolia*), *Euodia ailanthifolia* (*E. ailanthifolia*), *Euodia fargesii* (*E. fargesii*), *Euodia fraxinifolia* (*E. fraxinifolia*), *Euodia sutchuenensis* (*E. sutchuenensis*), *Euodia daniellii* (*E. daniellii*), and *Euodia delavayi* (*E. delavayi*)) were systematically examined using the LC-IT-TOF/MS detection method, based on untargeted metabolomics, with the goal of identifying the distinctive metabolites among these species. A progressive analysis of indole quinazoline alkaloids in *Euodia* was carried out, as well as the simultaneous determination of their in vitro anti-tumor activity and antimicrobial activity to reveal the different species of *Euodia* plant secondary metabolites and their related activities. This strategy can be used to guide the rapid acquisition of special metabolites at a high yield, and to develop the prospect of developing other plant compounds from *Euodia* into drugs or fungicidal pesticides. Therefore, we could improve the utilization efficiency of plant resources in *Euodia*, and further explore the potential of these species as medicinal plants or for discovering new drugs.

## 2. Results and Discussion

### 2.1. LC-IT-TOF/MS Profiles of 11 Euodia Species

**Figure 1:**
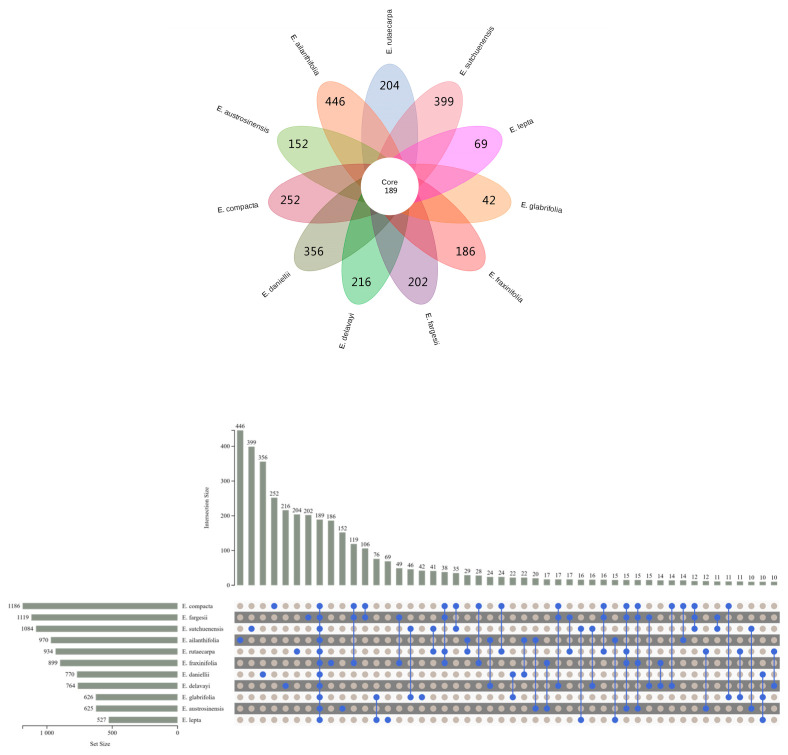
Venn diagram for putatively annotated metabolites in 11 *Euodia* species.

The TIC map was converted into a peak list and Wayne analysis was performed (Figure 1). It was found that *E. sutchuenensis*, *E. ailanthifolia*, and *E. daniellii* had a higher number of characteristic metabolites than other species, and *E. daniellii* had a lower compound abundance than the other two species. This indicated that the proportion of characteristic metabolites in this species was higher than that in the other two species. *E. lepta* and *E. glabrifolia* had fewer species characteristics, and the number of compounds detected in *E. lepta* was also the lowest. In this study, the compound richness of *E. rutaecarpa* was not the highest, but in an intermediate position. The designation of *E. rutaecarpa* as a pharmacopeia species is not due to its compound abundance and the proportion of characteristic compounds. Therefore, it is necessary to analyze other species and look for varieties similar to *E. rutaecarpa*, so as to develop and utilize them. To interpret these findings better, a further analysis of the LC-IT-TOF/MS results is essential.

### 2.2. Metabolome Analysis of Euodia Species

Numerous studies on *E. rutaecarpa* have shown that indole quinazoline alkaloids are the characteristic constituents. In contrast, long-side-chain quinolone alkaloids and citrulline analogs are the main chemical constituents of *E. rutaecarpa*. In addition, evodiamine, rutaecarpine, and limonin are important indicators for testing the quality of “Wu-Zhu-Yu” herbs according to the Chinese Pharmacopoeia. A total of 5949 peaks were detected by untargeted LC-IT-TOF/MS metabolomics. This study detected 1186 metabolites in *E. compacta*, of which 1090 metabolites were unknown and 96 were putatively annotated. A total of 1119 metabolites were detected in *E. fargesii*, of which 1032 metabolites were unknown and 87 were putatively annotated. A total of 1084 metabolites were detected in *E. austrosinensis*, of which 957 metabolites were unknown and 75 were putatively annotated. A total of 934 metabolites were detected in *E. rutaecarpa*, of which 816 metabolites were unknown and 118 were putatively annotated. A total of 899 metabolites were detected in *E. fargesii*, of which 812 metabolites were unknown and 87 were putatively annotated. A total of 770 metabolites were detected in *E. daniellii*, of which 721 metabolites were unknown and 49 were putatively annotated. A total of 764 metabolites were detected in *E. delavayi*, of which 726 metabolites were unknown and 38 were putatively annotated. A total of 626 metabolites were detected in *E. glabrifolia*, of which 535 metabolites were unknown and 94 were putatively annotated. A total of 625 metabolites were detected in *E. austrosinensis*, of which 550 metabolites were unknown and 75 were putatively annotated. A total of 527 metabolites were detected in *E. lepta*, of which 484 metabolites were unknown and 43 were putatively annotated. To gain insights into the metabolite profiles of these 11 species of *Euodia*, 192 putatively annotated metabolites were selected for qualitative and quantitative studies, including alkaloids, terpenoids, flavonoids, and phenolic acids (Table S1).

**Figure 2:**
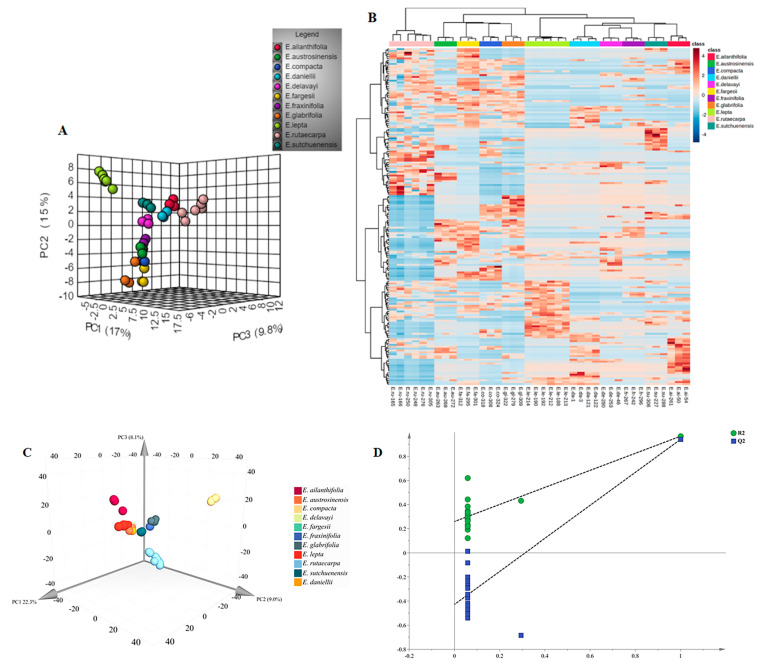
The PCA and heatmap clustering between *Euodia* species. (**A**) PCA analysis of all metabolites identified from *Euodia* species. (**B**) Heatmap clustering showing correlations among *Euodia* species samples based on global metabolic profiles. The color indicates the level of accumulation of each metabolite, from low (blue) to high (red). The *Z*−score represents the deviation from the median in standard deviation units. (**C**) PLS−DA analysis of all metabolites identified from *Euodia* species. (**D**) Permutations Plot for the PLS−DA model.

For the preliminary assessment of the differences between the metabolic profiles of these 11 species of *Euodia*, a principal component analysis (PCA) was conducted on the metabolic data. The results of the PCA showed that the first three components (PC1–PC3) accounted for 17, 15, and 9.8% of the data variance, respectively (Figure 1). The *E. lepta* samples were well differentiated, forming a succinct group in the upper-right corner of the figure. Moreover, the samples of *E. daniellii*, *E. ailanthifolia*, *E. austrosinensis*, and *E. delavayi* were clustered in the middle of the PCA plot and were spatially closer to *E. rutaecarpa*. In addition, the other samples were clustered just below *E. rutaecarpa* to the left. The heat map shows the association between all the metabolites of the 11 species, with the differences between *E. lepta* and the other species being the largest in terms of chemical composition, relative to the mean; *E. rutaecarpa, E. compact, E. austrosinensis, E. glabrifolia,* and *E. fargesii* were discovered to have a higher relative content of chemical compounds compared to other species. Additionally, these species exhibited a close relationship with each other, while demonstrating minimal overlap with the other species (Figure 2B). 

In the partial least squares discriminant analysis (PLS-DA), we employed a principal component analysis to reduce the high-dimensional data to lower dimensions and establish an optimal hyperplane for classification [29]. By performing suitable data pre-processing and model optimization, PLS-DA can enhance the accuracy and dependability of the classification results (Figure 2C). The Permutations Plot helps to evaluate the authenticity of the current PLS-DA model (Figure 2D). When all the green *R*^2^ values to the left are lower than the *R*^2^ value of the original point to the right, it further confirms the credibility of the original model [30]. The results of the modeling analysis were as follows: with a cumulative explanatory power parameter *R*^2^*Y* (cum) it was 0.971, with *R*^2^*X* (cum) it was 0.835, and with the predictive power parameter *Q*^2^ (cum) it was 0.947. Principal components 1 to 3 explain 39.96% of the variables. The model displays a distinct separation that is likely in line with the findings reported by unsupervised analyses. 

### 2.3. Differentially Accumulated Metabolites between Euodia Species 

In this study, a univariate statistical analysis method was used to analyze the metabolite profiles of 11 species of *Euodia*. The fold change difference and *p*-value were combined to identify the data with large changes and statistical significance, and the final results are presented in the volcanic map. As a variety of medicinal materials in the Pharmacopoeia of the People’s Republic of China, *E. rutaecarpa* has been widely developed and utilized, while the other 10 species have not been developed, so *E. rutaecarpa* was selected as the comparison group. The relevant data were processed, and the variation between *E. rutaecarpa* and the other species was illustrated using volcano plots.

Between *E. rutaecarpa* and *E. austrosinensis*, differences in 49 metabolites were identified (21 upregulated and 29 downregulated). A similar trend was observed between *E. rutaecarpa* and *E. compacta*, for which differences in 62 metabolites were identified (30 upregulated and 27 downregulated). Differences in 60 metabolites were detected between *E. rutaecarpa* and *E. fargesii* (34 upregulated and 26 downregulated). *E. rutaecarpa* and *E. glabrifolia* had differences in 64 metabolites (36 upregulated and 29 downregulated). Differences in 50 metabolites were identified between *E. rutaecarpa* and *E. ailanthifolia* (15 upregulated and 34 downregulated). Similarly, differences in 35 metabolites (11 upregulated and 25 downregulated) were observed between *E. rutaecarpa* and *E. daniellii*. Differences in 21 metabolites were detected (3 upregulated and 18 downregulated) between *E. rutaecarpa* and *E. delavayi*. Between *E. rutaecarpa* and *E. fraxinifolia*, differences in 26 metabolites were detected (5 upregulated and 20 downregulated). Between *E. rutaecarpa* and *E. lepta*, differences in 65 metabolites were detected (28 upregulated and 38 downregulated). Finally, *E. rutaecarpa* and *E. sutchuenensis* had differences in 28 metabolites (6 up-regulated and 22 down-regulated).

### 2.4. Specific Characteristics of Metabolites in Euodia Species

**Figure 3:**
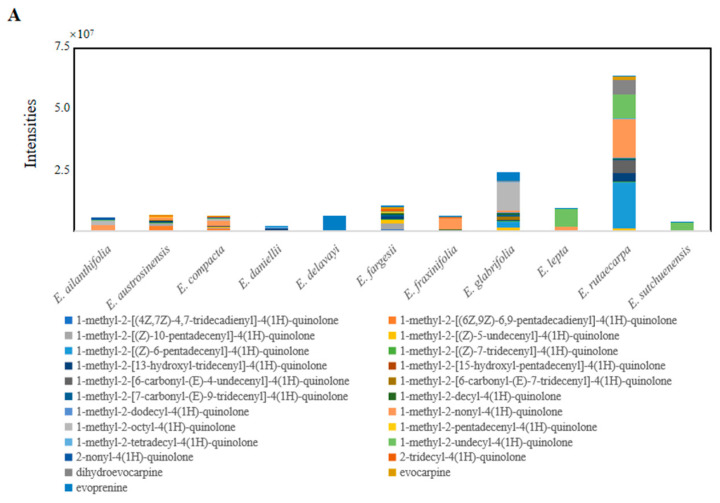

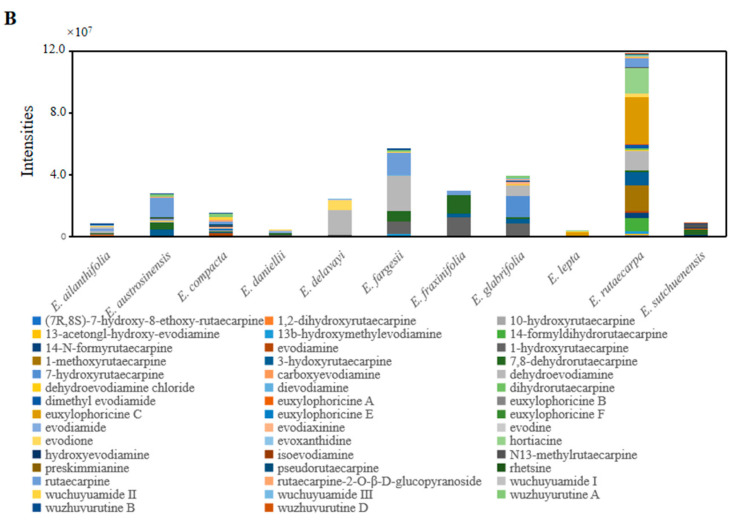
(**A**) Indole quinazoline alkaloids in putatively annotated metabolites from 11 species of *Euodia*; (**B**) indole quinazoline alkaloids in putatively annotated metabolites from 11 species of *Euodia*.

Quinolone and indole quinazoline alkaloids are unique components of *E. rutaecarpa* and are of medical value. The MS and MS/MS results obtained using ion trap mass spectrometry were used to analyze the content of these alkaloids (Tables S2 and S3). According to the findings of a comparative study on the alkaloid content of *Euodia* species, it was determined that *E. rutaecarpa*, *E. austrosinensis*, and *E. compacta* showed high levels of alkaloid accumulation among the species analyzed (Figure 3A). Comparing the different types of alkaloids, it was found that *E. rutaecarpa* had the most species of quinolone alkaloids, followed by *E. compacta* and *E. glabrifolia*. In descending order of alkaloid content, the species were *E. rutaecarpa*, *E. compacta*, *E. glabrifolia*, *E. austrosinensis*, *E. austrosinensis*, *E. glabrifolia*, *E. fargesii*, and *E. sutchuenensis* (Figure 3B). Among the indole quinazoline alkaloid types, *E. rutaecarpa* had the highest abundance, followed by *E. compacta* and *E. austrosinensis*. The accumulation of total alkaloids was most significant in *E. rutaecarpa*, *E. austrosinensis*, and *E. compacta*, compared with the other species.

### 2.5. Biological Activity Determination

#### 2.5.1. Anti-Tumor Activity

**Figure 4:**
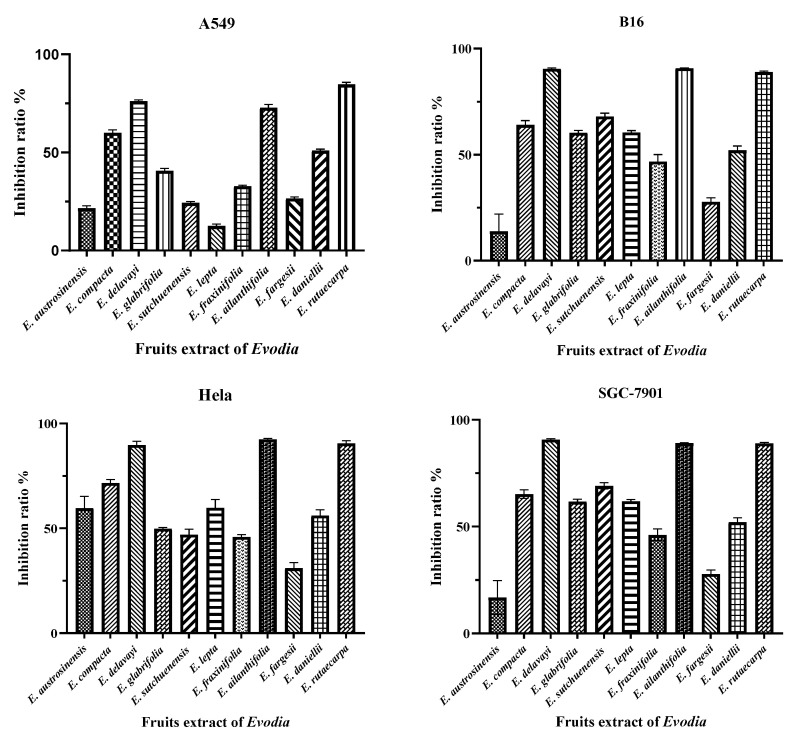
The inhibitory effect of the dichloromethane extract of 11 species of *Euodia* on tumor cell lines.

**Figure 5:**
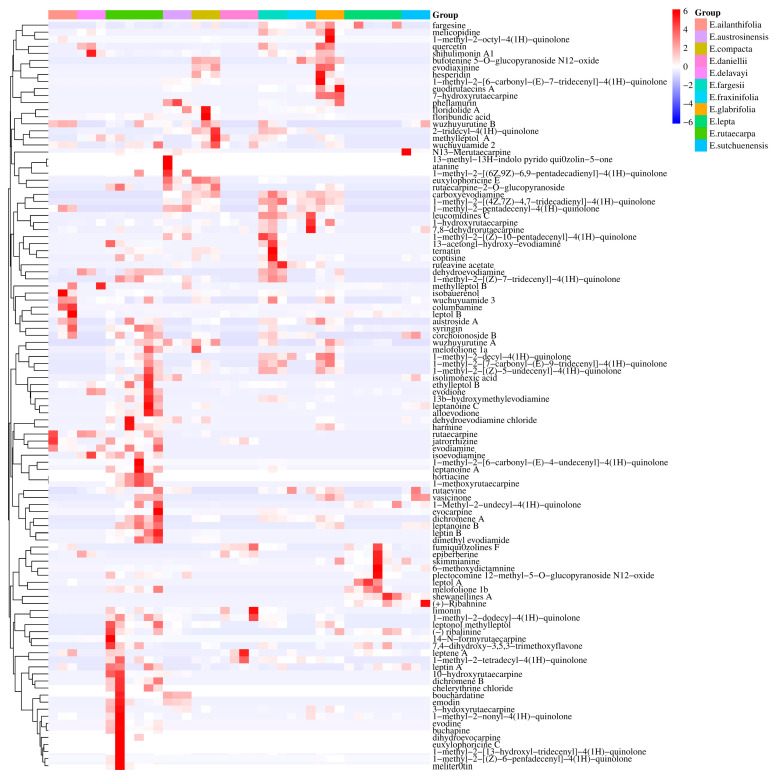
Cluster heat map of indole quinazoline alkaloids in 11 species of *Euodia.*

Subsequently, the functional analysis of the 11 species of *Euodia* was carried out to determine the anti-tumor properties of *Euodia* fruit in vitro. In the cell-based assays, the anti-tumor activity depended on the variety of *Euodia*. According to a study that separated *Euodia* plant compounds in the laboratory, it was concluded that the indole quinazoline alkaloids were mainly concentrated in the dichloromethane site [31]. Therefore, the anti-tumor experiments were carried out using the dichloromethane extract of *Euodia* fruits. According to the anti-tumor results, *E. delavayi*, *E. ailanthifolia*, and *E. rutaecarpa* had strong cytotoxic effects on the A549, B16, Hela, and SGC-7901 cell lines, and the inhibition rate exceeded 75% (Figure 4). The IC_50_ values of the three species are shown in Table 1. A cluster analysis of the putatively identified indole quinazoline alkaloids revealed that the evodiamine, rutaecarpine, and jatrorrhizine contents were the highest among the three species (Figure 5). According to previous studies, evodiamine and rutaecarpine have significant cytotoxicity against the A549 [32,33,34], Hela [35,36], B16 [37,38], and SGC-7901 [39,40,41] cell lines. Jatrorrhizine and its derivatives have great potential as anti-tumor compounds [42,43]. Therefore, we speculate that the anti-tumor activity of these three species may be due to the accumulation of evodiamine, rutaecarpine, and jatrorrhizine, which is basically consistent with the prescribed medicinal ingredients of *E. rutaecarpa*.

#### 2.5.2. Antibacterial Activity

**Figure 6:**
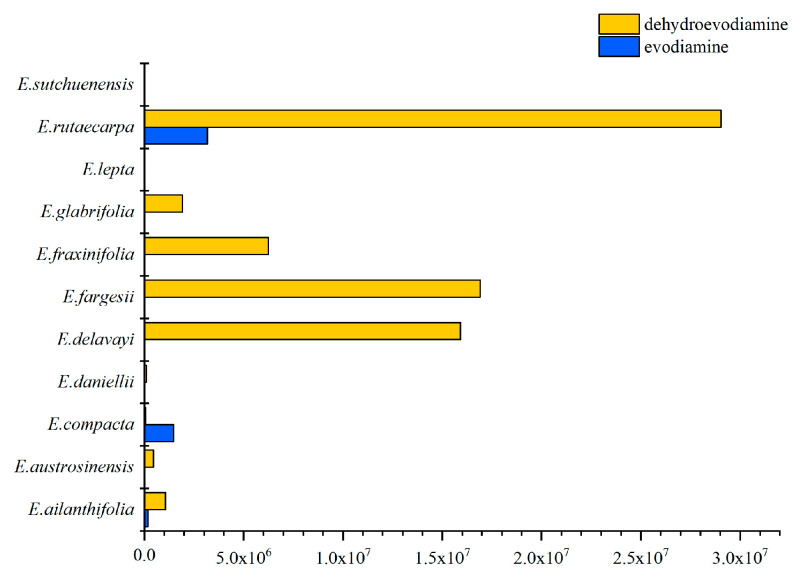
Evodiamine and dehydroevodiamine content distribution.

The activity of indole quinazoline alkaloids against plant pathogenic fungi and bacteria was verified to determine the biological characteristics of *Euodia*’s in vitro antimicrobial activity. We carried out a PLS model analysis on the putatively annotated metabolites to obtain Variable Importance in Projection (VIP) values for the metabolites, and we categorized metabolites with a VIP value > 1.5 as having a significant influence on sample classification or prediction [44]. From the compounds that were isolated in the laboratory, seven indole quinazoline alkaloids with differential expression were finally obtained (purity > 98%). The seven differentially expressed compounds were rutaecarpine, evodiamine, dihydroevocarpine, dehydroevodiamine, evodiaxinine, wuzhuyurutine B, and wuchuyuamide 3. It is worth noting that rutaecarpine and evodiamine are listed as active ingredients in traditional Chinese medicine in the Pharmacopoeia of the People’s Republic of China 2020 Edition. Through the preliminary screening of these compounds’ activity against plant pathogenic fungi and disease-resistant probacteria, it was found that the antibacterial activity in solutions of these alkaloids was good, especially in evodiamine, which had a good inhibitory effect on a variety of pathogenic fungi. The inhibition rate of dehydroevodiamine against *Xanthomonas oryzae* pv. *oryzae* was as high as 84.20%, and the inhibition rate of evodiamine against *Xanthomonas oryzae* pv. *oryzae* was also over 10% (Table 2). Dehydroevodiamine is abundant in *E. rutaecarpa*, *E. fargesii*, and *E. delavayi*, and there are small amounts in *E. fraxinifolia*, *E. ailanthifolia*, and *E. austrosinensis*. These *Euodia* plants can be considered for the development of pesticides against plant pathogenic bacteria. Evodiamine is mainly found in *E. rutaecarpa* and *E. compacta*. Both species can be developed as plant-derived pesticides against plant pathogenic fungi (Figure 6).

Through the application of metabolomics, it is possible to forecast the differences in the distribution of different chemical components between species, as well as identify the distribution of certain chemical components based on the growing conditions of a given species. In recent years, numerous studies have used metabolomic analysis for the analysis of differential plant secondary metabolites in the family Rutaceae, including *Pilocarpus*, *Citrus*, and *Zanthoxylum*. Moreover, a study on *Pilocarpus pennatifolius* showed that intensive greenhouse and field trials are important for determining the production of alkaloids and phenolics, as well as the associated environmental variables [45]. Blood oranges (*Citrus sinensis* cv. Tarocco) gave convincing evidence that phenylpropanoid metabolism may be triggered, and that metabolic flux may be switched to the synthesis of lignin precursors instead of flavonoids in vesicles during the collapse of blood oranges [46]. The varieties of *Z. bungeanum* have diverse metabolite compositions, with a predominance of phenolic substances, flavonoids, and phenolic acids. These compounds are especially abundant in July in variants of *Z. bungeanum* Maxim [47].

This study presents the first LC-IT-TOF/MS-based metabolomic analysis to compare the chemical compositions of 11 different species of *Euodia*. The environmental aspects need to be considered when investigating the factors affecting plant secondary metabolites. This is because plant–environment interactions, plant genotypes, and genotype–environment interactions affect the expression of chemical constituents in plants. The novelty of this study is that it provides a new perspective that can be used to better understand the differences in chemical composition between different species. At the same time, it provides a scientific reference for further exploring the full utilization of *Euodia* resources.

Over 90 alkaloids have been identified in *E. rutaecarpa*, with quinolone and indole quinazoline alkaloids accounting for the largest proportions. These compounds provide a strong support for our study of the alkaloid content and abundance in the 11 species of *Euodia*. The quinolone and indole quinazoline alkaloids are two important classes of *N*-based heterocyclic aromatic compounds [48]. Related studies have shown that the cytotoxicity of quinolone alkaloids may be related to their longer side chains and unsaturated carbonyl groups. Interestingly, another study found that the number of double bonds and the length of their aliphatic side chain at C-2 of quinolone alkaloids may affect their ability to kill cancer cells (HL-60, N-87, H-460, and Hep G2 cell lines) [49]. Although quinolone alkaloids are mainly found in the family Rutaceae, they have also recently been identified in endophytic fungal marine creatures [50]. However, evodiamine and rutaecarpine are alkaloids that are independent of *E. rutaecarpa* and their structural features put them in the indole quinazoline class. Notably, a previous study indicated that indole quinazoline alkaloids with a higher cytotoxicity have substituted phenolic hydroxyl groups on ring *E* [51]. This study mainly discussed the distribution and function of indole quinazoline alkaloids in vitro, which provided support for the development of indole quinazoline alkaloid-rich *Euodia* resources. The next step can focus on the abundant quinolone alkaloids in *Euodia*.

The results of this study showed that in terms of overall secondary metabolites, among these 11 species, *E. compacta*, *E. austrosinensis*, *E. glabrifolia*, and *E. fargesii* were more similar to *E. rutaecarpa* in terms of chemical composition, whereas *E. lepta* was different from *E. rutaecarpa*. A further analysis of the content and abundance of quinolone and indole quinazoline alkaloids revealed that *E. compacta*, *E. austrosinensis*, *E. glabrifolia*, and *E. fargesii* had higher contents or abundances of these alkaloids. Therefore, broadening the scope of research on *Euodia*, especially *E. compacta*, *E. austrosinensis*, *E. glabrifolia*, and *E. fargesii*, is essential to identifying additional quinolone and indole quinazoline alkaloids, such as dehydroevodiamine, evodiamine, and rutaecarpine. In addition, according to the results of the in vitro functional analysis, *E. rutaecarpa*, *E. delavayi*, and *E. ailanthifolia* could be further developed as new sources of anticancer raw materials. *E. fraxinifolia*, *E. ailanthifolia*, *E. austrosinensis*, and *E. delavayi* can be developed and utilized as plant sources of pesticides.

Therefore, this study used an innovative metabolomics strategy to study *Euodia*. First, differences in metabolite levels were characterized in 11 species using PCA, clustering, and volcano maps. Subsequently, differences in the distribution and in vitro activity of quinolone and indole quinazoline alkaloids in these species were investigated in depth. The main focus of this study was *E. rutaecarpa*, and there was no comprehensive analysis of *Euodia*. This novel strategy can be extended to other species of *Euodia*, particularly when *E. rutaecarpa* materials are limited. This approach facilitates having an availability of abundant medicinal plants for plant-derived drug discovery, enhances the utilization of botanical resources of species in *Euodia* that are not in the Chinese Pharmacopoeia, and creates the possibility for the discovery of lead compounds with developmental promise.

## 3. Materials and Methods

### 3.1. Solvents and Reagents

The methanol and dichloromethane used in the chromatography sample preparation were all analytical-grade chemicals purchased from Merck (Darmstadt, Germany) and Roe Scientific Inc. (Newark, DE, USA). Ultrapure water was obtained using a Miaozhiyi MZY-U device (Miaozhiyi Electronic Technology Co., Ltd., Nanjing, China).

### 3.2. Plant Material

Fruits from 11 species of *Euodia* (*E. lepta*, *E. rutaecarpa*, *E. compacta*, *E. austrosinensis*, *E. glabrifolia*, *E. ailanthifolia*, *E. fargesii*, *E. fraxinifolia*, *E. sutchuenensis*, *E. daniellii*, and *E. delavayi*) were collected in July and September between 2016 and 2018 in several locations. In each species distribution area, 3 to 6 trees were randomly selected and 10 g samples were collected at the same height in the four directions of the tree. Voucher specimens were housed at the Herbarium of the Institute of Botany, Jiangsu Province, and the Chinese Academy of Sciences. Based on the Flora Reipublicae Popularis Sinicae, professors Changqi Yuan and Qizhi Wang verified the plant materials based on morphological characteristics (Editorial Committee of the Chinese Flora 1997). The fresh plant materials were dried in the shade at room temperature for two weeks. Table 3, Tables S4 and S5 list the samples used in this study and their herbarium numbers.

### 3.3. Sample Preparation

Fresh fruits from *E. lepta* and *E. rutaecarpa* were collected from 6 different geographical locations, and the plant materials from each geographical location were uniformly mixed to create one biological replicate; a total of 6 biological replicates were used. The other species were collected at 3 different geographical locations, with a total of 3 biological replicates. Each sample of the dried plant material (approximately 0.52 g) was precisely weighed, placed in a conical container, and the metabolites were extracted with 5 mL of the solvent using an ultrasonic bath at room temperature for 10 min (40 kHz, 100 W). The vessel was then centrifuged at room temperature for 5 min at 10,000 rpm and the supernatant was collected. The residue was extracted with 5 mL of methanol twice, using an ultrasonic generator (40 kHz, 100 W) for 10 min. The supernatant was collected three times and combined before being dried at 45 °C in a vacuum using a rotating evaporator (Buchi Rotavapor R-210 system, BÜCHI Labortechnik AG, Flawil, Switzerland). The dried extracts were dissolved in 5 mL methanol (HPLC grade). Before analysis, the material was filtered through a 0.45 μm PVDF syringe filter. 

### 3.4. LC-IT-TOF/MS Apparatus and Conditions

The samples were analyzed using an LC-IT-TOF/MS system (Shimadzu, Tokyo, Japan). The LC experiments were conducted on a Shimadzu (Kyoto, Japan) HPLC system consisting of an LC-10AD binary pump, DGU-14A degasser, SIL-20AC autosampler, and a CTO-20AC column oven. Chromatographic separation was performed using an Agilent C18 column (Poroshell 120, 3.0 mm × 50 mm, 2.7 m, Agilent Technologies, Santa Clara, CA, USA) and a Poroshell 120 oven at a temperature of 35 °C. The gradient elution algorithm used a mobile phase composed of solvents A (methanol) and B (water containing 0.1% formic acid), running at a flow rate of 0.3 mL/min with 20:80 A/B for the first 2 min, followed by a linear gradient of 20:80–80:20 A for 48 min, followed by 100% B for 30 min. Subsequently, a 20 min re-equilibration process was performed between each run. For each sample, 5 µL of fruit extract was injected.

The mass detection was carried out using a Shimadzu ion trap/time-of-flight hybrid mass spectrometer (IT-TOF/MS) (Shimadzu, Kyoto, Japan) equipped with an electrospray ionization source. The operating settings were as follows: positive ion electrospray, nebulizing gas (N_2_) flow rate of 10 L/min, drying gas (N_2_) pressure of 172 KPa, and argon as the collision gas. The collision energy was set at 50% and the ion accumulation time was fixed at 30 ms. For TOF/MS accuracy calibration, a sodium trifluoroacetate solution was used as a reference sample. The LCMS Solution ver. 3.6 software was used to capture and process the data (Shimadzu, Tokyo, Japan).

### 3.5. Method Validation

Retention time stability, linearity, limit of detection (LOD), limit of quantification (LOQ), precision, analytical accuracy, and sample stability were all used to verify the LC-IT-TOF/MS analysis method for the collected samples. A total of 0.80 mg of evodiamine powder was precisely weighed, placed in a 10 mL volumetric bottle, and dissolved with methanol to obtain a control solution of evodiamine. A 2 mL volume of the evodiamine solution was diluted to obtain 6 different concentrations (80 μg/mL, 40 μg/mL, 20 μg/mL, 10 μg/mL, 5 μg/mL, and 2.5 μg/mL), which were tested using the method described in Section 3.4. The results were plotted with concentration on the abscissa and the peak area on the y coordinates; the linear regression analysis and the signal-to-noise ratios (S/N) were approximately 3 and 10, respectively, and were used to determine the limit of detection (LOD) and the limit of quantification (LOQ). Precision experiment: A 1 mL volume of the evodiamine reference solution was injected 6 times according to the method in Section 3.4, the peak area was recorded, and the RSD value was calculated. The chromatographic and retention time stabilities were calculated from five sequential measurements of the sample solution of *E. rutaecarpa*. Variations within and between days were used to assess the accuracy of the LC-IT-TOF/MS results. The *E. rutaecarpa* sample solution was analyzed five times in 1 d to determine the intra-day precision and thrice over the course of 2 d to determine the inter-day precision. To verify the assay repeatability, six replicate solutions made from the same sample (*E. rutaecarpa*) were examined. The results are shown in Table S6.

### 3.6. Statistical Analysis of Metabolite Data

Using MetaboAnalyst 5.0 (https://dev.metaboanalyst.ca/ (accessed on 11 June 2022)), the chromatograms obtained from all samples were processed for LC-MS and multivariate data analysis (MVDA) [52]. Data filtering using the relative standard deviation was used to replace missing values with a minimum of one-fifth of the positive values for the related variables. Pareto scaling, log transformation, and normalization using the median were included in the normalization procedure. The metabolites were structurally annotated with accurate m/z values, associated adjuncts, and MS/MS characteristic fragment data, and were matched to a database KEGG (https://www.genome.jp/kegg/compound/ (accessed on 6 August 2022)), METLIN (https://metlin.scripps.edu/ (accessed on 22 August 2022)), HMDB (https://hmdb.ca/ (accessed on 21 September 2022)) and combined with a self-built database for putative annotation. In addition, previous studies on the chemical components of *E. rutaecarpa* were used for the LC/MS-based identification of secondary metabolites. This was performed using Excel 2010 (Microsoft, Redmond, WA, USA). Multivariate statistical analysis was performed using Metware Cloud, a free online platform for data analysis (https://cloud.metware.cn (accessed on 6 November 2023)), and SIMCA 14.1.

### 3.7. Determination of Biological Activities of Euodia Extracts

#### 3.7.1. Cytotoxic Activity

The cytotoxic activity of the extracts on 4 cell lines was determined by MTT colorimetry. A total of 0.5 g of MTT was dissolved in 100 mL of phosphate-buffered saline (PBS), filtered with a 0.22 μm filter membrane to remove any bacteria in the solution, and stored at 4 °C away from light. All four cell lines used in this study were tumor cell lines (SGC-7901 human gastric cancer cells, B16 melanoma cells, Hela cervical cancer cells, and A549 human lung adenocarcinoma cells; Shanghai Cell Bank, Chinese Academy of Sciences). SGC-7901 cells were grown in an RPMI 1640 medium supplemented with 10% fetal bovine serum, penicillin (100 U/mL), and streptomycin (100 µg/mL) (Gibco, Grand Island, NY, USA). B16, Hela, and A549 cells were grown in a high-glucose DMEM medium supplemented with 10% fetal bovine serum, penicillin (100 U/mL), and streptomycin (100 µg/mL) (Gibco, America). The cells were harvested at the log phase of growth and then seeded into 96-well plates (100 µL/well at a density of 5 × 10^5^ cells/mL). After a 24 h incubation at 37 °C and 5% CO_2_ to allow for cell attachment in a Thermo-6500 CO_2_ Cell incubator (Thermo, America), the cultures were exposed to the extracts from the different species at various concentrations (100 μg/mL, 50 μg/mL, 25 μg/mL, 12.5 μg/mL, and 6.25 μg/mL) for 72 h. A positive control (cisplatin), negative control (cell culture without extract), and blank control (culture only) were also tested. Then, the MTT solution was added (10 µL/well) and the plates were incubated for 4 h at 37 °C and 5% CO_2_. Absorption at 450 nm was measured with an Infinite M200 Microplate Reader (TECAN, Morrisville, NC, USA), and the IC_50_ value was recorded as the concentration at which 50% of the cells were dead. All assays were performed in triplicate, and the results are expressed as the mean ± standard deviation. The samples for cytotoxicity detection were leached using methylene chloride (for alkaloid enrichment) from the plant materials. The dichloromethane fraction was freeze-dried and then dissolved in DMSO to prepare a stock liquor with a concentration of 10 mg/mL for use.

#### 3.7.2. Activity against Plant Pathogenic Fungi

The antifungal effects of the tested compounds against 5 plant fungi (*Sclerotinia sclerotiorum*, *Rhizoctonia solani*, *Fusarium graminearum*, *Botrytis cinerea*, and *Colletotrichum gloeosporioides*) were measured by the mycelium growth rate method. The tested compounds were rutaecarpine, evodiamine, dihydroevocarpine, dehydroevodiamine, evodiaxinxine, wuzhuyurutine B, and wuchuyuamide Ⅲ, which we isolated from the dried near-ripe fruit of *E. rutaecarpa* as ethanol extracts with a purity ≥ 90%. The experimental protocol was described in a previous study [53]. In brief, the PSA medium was heated and melted, cooled to about 55 °C, and 400 μL of the test sample stock solution or DMSO (solvent control) was added to every 200 mL of the medium; then, the medium was transferred into Petri dishes with a diameter of 9 cm to make drug-containing plates. PSA plates with fungi with strong mycelium growth, and grown in a 25 °C incubator, were used to inoculate the drug-containing plates and a solvent control group (DMSO) plate, which were then cultured in a 25 °C incubator. The inhibition rates of the different extracts on mycelial growth were determined by measuring and calculating the colony diameter. The experiment was performed three times, and the data were calculated using data processing software.

#### 3.7.3. Activity against Plant Pathogenic Bacteria

The bacteriostatic activity of the tested compounds against *Xanthomonas oryzae* pv. *oryzae* was determined by nephelometry. The tested compounds were rutaecarpine, evodiamine, dihydroevocarpine, dehydroevodiamine, evodiaxinxine, wuzhuyurutine B, and wuchuyuamide Ⅲ, which we isolated from the dried near-ripe fruit of *E. rutaecarpa* as ethanol extracts with a purity ≥ 90%. The experimental protocol was described in a previous study [54]. The nutrient broth (NB) was prepared using the same method described in the literature [55]. A 50 μL volume of a 5000 μg/mL solution of the test sample or DMSO (solvent control) was added into 25 mL of the medium and mixed well to obtain a drug-containing medium. Then, bacteria in the logarithmic growth stage were selected as the bacteria source, and the NB medium was used to adjust the OD_600_ to 0.02. The bacterial solution (100 μL) was carefully added to the drug-containing medium. The mixture was thoroughly mixed to ensure proper integration. Meanwhile, a control group was established using DMSO. Finally, the drug-containing medium with the added bacteria was placed in a shaker at 28 ≥ (175 r/min) (Shanghai Cimo Medical instrument Co., Ltd, Shanghai, China). A spectrophotometer was used to measure the absorption value at a wavelength of 600 nm. The formula [(OD_control_ − OD_samples_)/OD_control_] × 100% was used to calculate the bacteriostatic rate. 

## 4. Conclusions

In this study, 11 *Euodia* fruit samples were prepared and analyzed using LC-IT-TOF/MS under the same conditions. The preliminary results showed that the metabolites of *E. lepta* differed significantly from those of the other species. Further studies revealed that the highest number and content of quinolone and indole quinazoline alkaloids were found in *E. rutaecarpa*, followed by *E. compacta*, *E. austrosinensis*, *E. glabrifolia*, and *E. fargesii*. Anti-tumor experiments showed that the alkaloids of *E. delavayi*, *E. ailanthifolia,* and *E. rutaecarpa* plant materials had obvious cytotoxicity effects on tumor cells. The metabolite analysis showed that these three plants may be rich in evodiamine, rutaecarpine, and jatrorrhizine. Antimicrobial activity assays have shown that species rich in evodiamine and dehydroevodiamine have the potential to be exploited as antibacterial agents. Through in-depth studies of the potential pharmacological effects, biosynthetic pathways, and key enzymes of quinolone and indole quinazoline alkaloids, the metabolomic strategy proposed in this study for *Euodia* is instructive. This study elucidates the chemical composition differences among *Euodia* species and better illustrates the biological activity among different species, paving the way for the industrial exploitation of *Euodia* resources.

## Figures and Tables

**Table 1:** Inhibitory concentration of 50% for cytotoxic activity.

	Cytotoxic Activity (IC_50_, µg/mL)			
Species	A549	B16	Hela	SGC-7901
*E. delavayi*	67.23 ± 1.52	50.37 ± 0.97	52.20 ± 1.12	51.87 ± 0.47
*E. ailanthifolia*	69.07 ± 2.31	51.23 ± 0.45	50.07 ± 0.53	53.25 ± 0.38
*E. rutaecarpa*	62.16 ± 1.44	53.15 ± 0.64	51.01 ± 1.35	51.21 ± 0.35
cisplatin ^a^	3.34 ± 0.12	2.44 ± 1.22	4.22 ± 1.92	3.12 ± 0.12

^a^ Positive control.

**Table 2:** Inhibition rate of test compounds against plant pathogenic fungi and bacteria.

Compounds	*Sclerotinia sclerotiorum* (10 μg/mL, %)	*Rhizoctonia solani* (10 μg/mL, %)	*Fusarium graminearum* (10 μg/mL, %)	*Botrytis cinerea* (10 μg/mL, %)	*Colletotrichum gloeosporioides* (10 μg/mL, %)	*Xanthomonas oryzae* pv*. Oryzae* (10 μg/mL, %)
rutaecarpine	8.20 ± 1.21	11.11 ± 1.11	17.78 ± 1.67	8.89 ± 4.44	13.33 ± 2.22	4.88 ± 2.51
evodiamine	40.00 ± 5.56	33.33 ± 2.78	22.22 ± 2.78	22.22 ± 3.89	37.77 ± 3.89	13.13 ± 1.24
dihydroevocarpine	0.56 ± 2.32	5.56 ± 1.11	4.24 ± 2.14	8.75 ± 4.44	3.70 ± 1.11	8.0 ± 2.11
dehydroevodiamine	6.67 ± 1.11	5.12 ± 4.12	13.33 ± 1.67	20.00 ± 3.89	20.00 ± 3.33	84.20 ± 6.87
evodiaxinxine	15.71 ± 1.21	8.91 ± 2.22	9.97 ± 1.67	2.70 ± 1.24	11.07 ± 3.33	1.16 ± 2.22
wuzhuyurutine B	10.12 ± 1.11	0.66 ± 0.54	4.79 ± 0.67	7.27 ± 3.33	4.20 ± 1.11	4.2 ± 1.11
wuchuyuamide III	10.90 ± 2.22	12.54 ± 1.67	17.87 ± 2.14	6.08 ± 2.22	18.70 ± 2.22	0.83 ± 1.24

**Table 3:** List of *Euodia* samples used in the study.

No.	Species	Sample Symbol	Collection Year	GPS Coordinates of Collection Site
1	*Euodia lepta* (*E. lepta*)	ELE20171008-A	8 October 2017	21°55′27.26″ N 101°15′27.44″ E
2	*Euodia lepta* (*E. lepta*)	ELE20171011-B	11 October 2017	22°50′7.36″ N 100°59′56.94″ E
3	*Euodia lepta* (*E. lepta*)	ELE20171017-C	17 October 2017	24°53′49.81″ N 110°57′51.90″ E
4	*Euodia lepta* (*E. lepta*)	ELE20160801-D	1 August 2016	18°42′17.72″ N 109°51′17.81″ E
5	*Euodia lepta* (*E. lepta*)	ELE20160801-E	1 August 2016	18°39′20.93″ N 109°54′29.38″ E
6	*Euodia lepta* (*E. lepta*)	ELE20160801-F	1 August 2016	18°40′28.84″ N 109°54′10.84″ E
7	*Euodia rutaecarpa* (*E. rutaecarpa*)	ERU20181024-A	24 September 2018	32°03′09.68″ N 118°50′03.20″ E
8	*Euodia rutaecarpa* (*E. rutaecarpa*)	ERU20160715-B	15 September 2016	26°16′12.57″ N 106°58′49.82″ E
9	*Euodia rutaecarpa* (*E. rutaecarpa*)	ERU20160727-C	27 August 2016	27°36′20.64″ N 105°50′46.37″ E
10	*Euodia rutaecarpa* (*E. rutaecarpa*)	ERU20160731-D	31 August 2016	25°54′0.55″ N 104°59′43.88″ E
11	*Euodia rutaecarpa* (*E. rutaecarpa*)	ERU20180721-E	21 September 2018	27°50′46.12″ N 109°14′29.63″ E
12	*Euodia rutaecarpa* (*E. rutaecarpa*)	ERU20180722-F	22 September 2018	27°13′16.53″ N 107°56′22.79″ E
13	*Euodia compacta* (*E. compacta*)	ECO20180521-A	21 August 2018	30°32′45.75″ N 114°25′18.06″ E
14	*Euodia compacta* (*E. compacta*)	ECO20180910-B	10 September 2018	30°15′10.26″ N 120°07′05.53″ E
15	*Euodia compacta* (*E. compacta*)	ECO20180910-C	10 September 2018	30°15′13.40″ N 120°07′02.75″ E
16	*Euodia austrosinensis* (*E. austrosinensis*)	EAU20181008-A	8 October 2018	21°55′27.26″ N 101°15′27.44″ E
17	*Euodia austrosinensis* (*E. austrosinensis*)	EAU20181017-B	17 October 2018	24°53′54.01″ N 110°59′19.65″ E
18	*Euodia austrosinensis* (*E. austrosinensis*)	EAU20180730-C	30 August 2018	21°55′27.26″ N 101°15′27.44″ E
19	*Euodia glabrifolia* (*E. glabrifolia*)	EGL20160801-A	1 August 2016	18°41′16.85″ N 109°43′44.21″ E
20	*Euodia glabrifolia* (*E. glabrifolia*)	EGL20160801-B	1 August 2016	18°40′51.09″ N 109°51′35.11″ E
21	*Euodia glabrifolia* (*E. glabrifolia*)	EGL20160801-C	1 August 2016	18°42′31.58″ N 109°50′28.71″ E
22	*Euodia ailanthifolia* (*E. ailanthifolia*)	EAI20181008-A	8 October 2018	21°55′27.26″ N 101°15′27.44″ E
23	*Euodia ailanthifolia* (*E. ailanthifolia*)	EAI20181011-B	22 October 2018	22°50′7.36″ N 100°59′56.94″ E
24	*Euodia ailanthifolia* (*E. ailanthifolia*)	EAI20180729-C	29 August 2018	22°46′21.55″ N 100°59′31.66″ E
25	*Euodia fargesii* (*E. fargesii*)	EFA20160910-A	10 September 2016	30°14′57.93″ N 120°07′06.80″ E
26	*Euodia fargesii* (*E. fargesii*)	EFA20160731-B	31 August 2016	25°47′13.18″ N 104°58′17.48″ E
27	*Euodia fargesii* (*E. fargesii*)	EFA20180518-C	18 September 2016	28°06′11.01″ N 113°01′55.86″ E
28	*Euodia sutchuenensis* (*E. sutchuenensis*)	ESU20160816-A	16 August 2016	29°04′20.90″ N 107°09′38.44″ E
29	*Euodia sutchuenensis* (*E. sutchuenensis*)	ESU20160817-B	17 August 2016	29°05′59.06″ N 107°10′06.24″ E
30	*Euodia sutchuenensis* (*E. sutchuenensis*)	ESU20180811-C	11 August 2018	29°04′51.28″ N 107°08′49.46″ E
31	*Euodia daniellii* (*E. daniellii*)	EDA20170916-A	16 September 2017	41°54′33.51″ N 123°36′01.37″ E
32	*Euodia daniellii* (*E. daniellii*)	EDA20170920-B	20 September 2017	34°42′49.50″ N 119°22′41.94″ E
33	*Euodia daniellii* (*E. daniellii*)	EDA20170924-C	24 September 2017	36°38′48.54″ N 117°01′18.37″ E
34	*Euodia daniellii* (*E. daniellii*)	EDA20170924-D	24 September 2017	36°38′46.09″ N 117°01′21.31″ E
35	*Euodia delavayi* (*E. delavayi*)	EDE20181024-A	24 October 2018	28°26′32.58″ N 98°54′49.57″ E
36	*Euodia delavayi* (*E. delavayi*)	EDE20180804-B	4 August 2018	26°52′10.29″ N 100°13′54.36″ E
37	*Euodia delavayi* (*E. delavayi*)	EDE20180810-C	10 August 2018	29°03′59.43″ N 107°08′43.13″ E
38	*Euodia fraxinifolia* (*E. fraxinifolia*)	EFR20180808-A	8 August 2018	24°45′51.16″ N 100°30′10.87″ E
39	*Euodia fraxinifolia* (*E. fraxinifolia*)	EFR20180802-B	2 August 2018	24°23′28.77″ N 100°46′47.38″ E
40	*Euodia fraxinifolia* (*E. fraxinifolia*)	EFR20180806-C	6 August 2018	25°27′45.18″ N 98°45′54.14″ E

## Data Availability

Research data are not shared.

## Supplementary Materials

The following supporting information can be downloaded at: https://www.mdpi.com/article/10.3390/molecules29051059/s1, Supplementary File S1: Table S1. 192 differential metabolites in *Evodia*; Supplementary File S2: Table S2. List of quinolone alkaloids; Supplementary File S3: Table S3. List of Indole quinazoline alkaloids; Supplementary File S4: Table S4. Plant material information supplement; Table S5. Collection time Environmental status; Table S6. Summary of method validation.
