# Supplementary material for: Comprehensive Analysis of 11 Species of Euodia (Rutaceae) by Untargeted LC-IT-TOF/MS Metabolomics and In Vitro Functional Methods
Source: Molecules. 2024 Feb 28;29(5):1059. doi: 10.3390/molecules29051059 (PMC10934029; doi:10.3390/molecules29051059)
Supplement: Supplementary file 1 [file molecules-29-01059-s001.zip › Supplementary file 1.pdf]

Table S1. 192 differential metabolites in *Evodia*

Class I	Class II	Compounds	Species	Molecular Weight	Rt (min)	Formula	Ionization model
Alkaloids	Indole quinazoline alkaloids	evoxanthidine	*E.rutaecarpa*	270.09549	1.02170	$C_{15}H_{11}NO_4$	$[M+H]^+$
Alkaloids	Indole quinazoline alkaloids	7,8-dehydrorutaecarpine	*E.ailanthifolia, E.austrosinensis, E.compacta, E.daniellii, E.fargesii, E.fraxinifolia, E.glabrifolia, E.rutaecarpa, E.sutchuenensis*	286.10168	9.32874	$C_{18}H_{11}N_3O$	$[M+H]^+$
Alkaloids	Indole quinazoline alkaloids	isoevodiamine	*E.delavayi, E.rutaecarpa*	288.09169	16.6449 3	$C_{18}H_{13}N_3O$	$[M+H]^+$
Alkaloids	Indole quinazoline alkaloids	pseudorutaecarpine	*E.compacta, E.delavayi*	288.10489	1.31414	$C_{18}H_{13}N_3O$	$[M+H]^+$
Alkaloids	Indole quinazoline alkaloids	rutaecarpine	*E.sutchuenensis*	288.10489	4.00941	$C_{18}H_{13}N_3O$	$[M+H]^+$
Alkaloids	Indole quinazoline alkaloids	wuzhuyurutine A	*E.austrosinensis, E.compacta, E.fargesii, E.glabrifolia, E.lepta, E.rutaecarpa*	290.08859	0.89975	$C_{17}H_{11}N_3O_2$	$[M+H]^+$
Alkaloids	Indole quinazoline alkaloids	dimethyl evodiamide	*E.ailanthifolia, E.austrosinensis, E.compacta, E.daniellii, E.delavayi, E.fargesii, E.glabrifolia, E.rutaecarpa*	290.13449	2.66598	$C_{18}H_{15}N_3O$	$[M+H]^+$

Class I	Class II	Compounds	Species	Molecular Weight	Rt (min)	Formula	Ionization model
Alkaloids	Indole quinazoline alkaloids	evodione	*E.daniellii, E.delavayi, E.glabrifolia, E.lepta, E.rutaecarpa*	293.10248	3.97998	$C_{16}H_{20}O_5$	$[M+H]^+$
Alkaloids	Indole quinazoline alkaloids	dimethyl evodiamide	*E.compacta, E.rutaecarpa*	294.14978	0.91878	$C_{18}H_{19}N_3O$	$[M+H]^+$
Alkaloids	Indole quinazoline alkaloids	dehydroevodiamine	*E.ailanthifolia, E.austrosinensis, E.compacta, E.daniellii, E.delavayi, E.fargesii, E.glabrifolia, E.rutaecarpa*	302.12189	28.06648	$C_{19}H_{15}N_3O$	$[M+H]^+$
Alkaloids	Indole quinazoline alkaloids	*N*13-methyl-rutaecarpine	*E.austrosinensis, E.compacta, E.rutaecarpa, E.sutchuenensis*	302.14679	80.23433	$C_{19}H_{15}N_3O$	$[M+H]^+$
Alkaloids	Indole quinazoline alkaloids	evodiamine	*E.ailanthifolia, E.delavayi, E.rutaecarpa*	304.10449	10.86117	$C_{19}H_{17}N_3O$	$[M+H]^+$
Alkaloids	Indole quinazoline alkaloids	10-hydroxyrutaecarpine	*E.austrosinensis, E.rutaecarpa*	304.10870	16.67472	$C_{18}H_{13}N_3O_2$	$[M+H]^+$
Alkaloids	Indole quinazoline alkaloids	1-hydroxyrutaecarpine	*E.ailanthifolia, E.austrosinensis, E.compacta, E.daniellii, E.delavayi, E.fargesii, E.fraxinifolia, E.glabrifolia, E.rutaecarpa, E.sutchuenensis*	304.10870	23.12527	$C_{18}H_{13}N_3O_2$	$[M+H]^+$
Alkaloids	Indole quinazoline	3-hydoxyrutaecarpine	*E.austrosinensis, E.compacta, E.fargesii, E.fraxinifolia, E.glabrifolia, E.rutaecarpa*	304.10945	15.01041	$C_{18}H_{13}N_3O_2$	$[M+H]^+$

Class I	Class II	Compounds	Species	Molecular Weight	Rt (min)	Formula	Ionization model
	alkaloids						
Alkaloids	Indole quinazoline alkaloids	rhetsine	*E.austrosinensis, E.fargesii*	304.11775	1.53560	$C_{19}H_{17}N_3O$	$[M+H]^+$
Alkaloids	Indole quinazoline alkaloids	wuzhuyurutine B	*E.ailanthifolia, E.austrosinensis, E.compacta, E.fargesii, E.rutaecarpa, E.sutchuenensis*	306.11029	17.20765	$C_{17}H_{11}N_3O_3$	$[M+H]^+$
Alkaloids	Indole quinazoline alkaloids	wuzhuyurutine D	*E.rutaecarpa, E.sutchuenensis*	306.12698	0.92159	$C_{17}H_{11}N_3O_3$	$[M+H]^+$
Alkaloids	Indole quinazoline alkaloids	evodiamide	*E.ailanthifolia, E.austrosinensis, E.daniellii, E.rutaecarpa*	308.11850	6.70353	$C_{19}H_{21}N_3O$	$[M+H]^+$
Alkaloids	Indole quinazoline alkaloids	14-*N*-formyrutaecarpine	*E.delavayi, E.rutaecarpa, E.sutchuenensis*	316.21741	16.42000	$C_{19}H_{13}N_3O_2$	$[M+H]^+$
Alkaloids	Indole quinazoline alkaloids	14-formyldihydrorutaecarpine	*E.austrosinensis, E.rutaecarpa, E.sutchuenensis*	318.12180	3.81744	$C_{19}H_{15}N_3O_2$	$[M+H]^+$
Alkaloids	Indole quinazoline alkaloids	1-methoxyrutaecarpine	*E.rutaecarpa*	318.12180	16.07302	$C_{19}H_{15}N_3O_2$	$[M+H]^+$
Alkaloids	Indole quinazoline	hortiacine	*E.rutaecarpa*	318.12180	34.16657	$C_{19}H_{15}N_3O_2$	$[M+H]^+$

Class I	Class II	Compounds	Species	Molecular Weight	Rt (min)	Formula	Ionization model
	alkaloids						
Alkaloids	Indole quinazoline alkaloids	1,2-dihydroxyrutaecarpine	*E.ailanthifolia, E.austrosinensis, E.daniellii, E.glabrifolia, E.rutaecarpa*	320.10068	16.35150	$C_{18}H_{13}N_3O_3$	$[M+H]^+$
Alkaloids	Indole quinazoline alkaloids	wuchuyuamide III	*E.ailanthifolia, E.compacta, E.delavayi, E.fargesii, E.glabrifolia, E.rutaecarpa, E.sutchuenensis*	324.12830	1.52935	$C_{18}H_{17}N_3O_3$	$[M+H]^+$
Alkaloids	Indole quinazoline alkaloids	evodine	*E.rutaecarpa*	330.15628	6.32866	$C_{18}H_{19}NO_5$	$[M+H]^+$
Alkaloids	Indole quinazoline alkaloids	euxylophoricine C	*E.glabrifolia, E.lepta, E.rutaecarpa*	332.08508	8.95835	$C_{19}H_{13}N_3O_3$	$[M+H]^+$
Alkaloids	Indole quinazoline alkaloids	euxylophoricine F	*E.rutaecarpa, E.sutchuenensis*	334.11554	4.13329	$C_{19}H_{15}N_3O_3$	$[M+H]^+$
Alkaloids	Indole quinazoline alkaloids	13β-hydroxymethylevodiamine	*E.fargesii, E.glabrifolia, E.rutaecarpa*	334.21158	52.97942	$C_{20}H_{19}N_3O_2$	$[M+H]^+$
Alkaloids	Indole quinazoline alkaloids	wuchuyuamide II	*E.ailanthifolia, E.compacta, E.daniellii, E.fargesii, E.glabrifolia, E.rutaecarpa*	336.11719	3.20547	$C_{19}H_{17}N_3O_3$	$[M+H]^+$

Class I	Class II	Compounds	Species	Molecular Weight	Rt (min)	Formula	Ionization model
Alkaloids	Indole quinazoline alkaloids	dehydroevodiamine chloride	*E.austrosinensis, E.compacta, E.glabrifolia, E.rutaecarpa, E.sutchuenensis*	339.11569	3.24757	$C_{19}H_{16}ClN_3O$	$[M+H]^+$
Alkaloids	Indole quinazoline alkaloids	carboxyevodiamine	*E.austrosinensis, E.compacta, E.fargesii, E.fraxinifolia, E.glabrifolia*	348.12769	2.24740	$C_{20}H_{17}N_3O_3$	$[M+H]^+$
Alkaloids	Indole quinazoline alkaloids	evodiaxinine	*E.compacta, E.glabrifolia*	316.16611	59.20076	$C_{20}H_{17}N_3O$	$[M+H]^+$
Alkaloids	Indole quinazoline alkaloids	euxylophoricine B	*E.glabrifolia*	346.09949	1.54164	$C_{20}H_{15}N_3O_3$	$[M+H]^+$
Alkaloids	Indole quinazoline alkaloids	euxylophoricine E	*E.austrosinensis, E.compacta, E.fargesii*	376.17229	1.93245	$C_{21}H_{17}N_3O_4$	$[M+H]^+$
Alkaloids	Indole quinazoline alkaloids	rutaecarpine-2-*O*-*β*-D-glucopyranoside	*E.austrosinensis, E.compacta, E.glabrifolia, E.rutaecarpa*	466.43848	2.80646	$C_{24}H_{23}N_3O_7$	$[M+H]^+$
Alkaloids	Indole quinazoline alkaloids	dievodiamine	*E.compacta, E.fargesii*	603.25208	66.77623	$C_{38}H_{30}N_6O_2$	$[M+H]^+$
Alkaloids	Indole quinazoline alkaloids	(7*R*,8*S*)-7-hydroxy-8-ethoxy-rutaecarpine	*E.sutchuenensis*	320.10068	34.27879	$C_{18}H_{13}N_3O_3$	$[M+H]^+$

Class I	Class II	Compounds	Species	Molecular Weight	Rt (min)	Formula	Ionization model
Alkaloids	Indole quinazoline alkaloids	hydroxyevodiamine	*E.ailanthifolia, E.compacta, E.glabrifolia, E.sutchuenensis*	320.13480	5.19086	$C_{19}H_{17}N_3O_2$	$[M+H]^+$
Alkaloids	Indole quinazoline alkaloids	wuchuyuamide I	*E.ailanthifolia, E.daniellii, E.delavayi*	352.14819	18.2863 8	$C_{19}H_{17}N_3O_4$	$[M+H]^+$
Alkaloids	Indole quinazoline alkaloids	preskimmianine	*E.compacta, E.fargesii, E.fraxinifolia, E.glabrifolia*	304.14200	56.9229 6	$C_{17}H_{21}NO_4$	$[M+H]^+$
Alkaloids	Indole quinazoline alkaloids	euxylophoricine A	*E.compacta, E.rutaecarpa, E.sutchuenensis*	348.12769	2.02716	$C_{20}H_{17}N_3O_3$	$[M+H]^+$
Alkaloids	Quinoline alkaloid	columbamine	*E.ailanthifolia, E.daniellii, E.fargesii, E.glabrifolia, E.lepta*	339.13519	7.70203	$C_{20}H_{20}NO_4$	$[M+H]^+$
Alkaloids	Quinoline alkaloid	chelerythrine chloride	*E.rutaecarpa*	384.14125	1.20036	$C_{21}H_{18}ClNO_4$	$[M+H]^+$
Alkaloids	Quinoline alkaloid	palmatine	*E.ailanthifolia, E.compacta, E.daniellii, E.delavayi, E.fargesii*	353.17938	61.0168 2	$C_{21}H_{22}NO_4$	$[M+H]^+$
Alkaloids	Quinolone alkaloids	atanine	*E.austrosinensis, E.daniellii, E.glabrifolia, E.rutaecarpa*	244.11909	1.16988	$C_{15}H_{17}NO_2$	$[M+H]^+$
Alkaloids	Quinolone alkaloids	1-methyl-2-nonyl-4(1*H*)-quinolone	*E.ailanthifolia, E.austrosinensis, E.fraxinifolia, E.glabrifolia, E.lepta, E.rutaecarpa*	286.09848	35.8903 2	$C_{19}H_{27}NO$	$[M+H]^+$
Alkaloids	Quinolone	bouchardatine	*E.austrosinensis, E.rutaecarpa*	290.08859	19.9148	$C_{17}H_{11}N_3O_2$	$[M+H]^+$

Class I	Class II	Compounds	Species	Molecular Weight	Rt (min)	Formula	Ionization model
	alkaloids				9		
Alkaloids	Quinolone alkaloids	1-methyl-2-decyl-4(1*H*)-quinolone	*E.fargesii, E.fraxinifolia, E.glabrifolia, E.rutaecarpa*	300.32364	17.1407 8	$C_{20}H_{29}NO$	$[M+H]^+$
Alkaloids	Quinolone alkaloids	1-methyl-2-[(*Z*)-5-undecenyl]-4(1*H*)-quinolone	*E.austrosinensis, E.compacta, E.daniellii, E.fargesii, E.glabrifolia, E.rutaecarpa*	312.22290	40.2957 9	$C_{21}H_{29}NO$	$[M+H]^+$
Alkaloids	Quinolone alkaloids	1-methyl-2-undecyl-4(1*H*)-quinolone	*E.austrosinensis, E.daniellii, E.fargesii, E.glabrifolia, E.rutaecarpa*	314.25079	62.7489 2	$C_{21}H_{31}NO$	$[M+H]^+$
Alkaloids	Quinolone alkaloids	1-methyl-2-[6-carbonyl-(*E*)-4-undecenyl]-4(1*H*)-quinolone	*E.fargesii, E.rutaecarpa*	326.24640	54.2973 7	$C_{21}H_{27}NO_2$	$[M+H]^+$
Alkaloids	Quinolone alkaloids	1-methyl-2-dodecyl-4(1*H*)-quinolone	*E.austrosinensis, E.daniellii, E.fargesii, E.glabrifolia, E.rutaecarpa*	328.07730	44.4380 5	$C_{22}H_{33}NO$	$[M+H]^+$
Alkaloids	Quinolone alkaloids	1-methyl-2-[(*Z*)-7-tridecenyl]-4(1*H*)-quinolone	*E.austrosinensis, E.fargesii, E.glabrifolia, E.rutaecarpa*	340.25179	60.6724 7	$C_{23}H_{33}NO$	$[M+H]^+$
Alkaloids	Quinolone alkaloids	evocarpine	*E.austrosinensis, E.fargesii, E.glabrifolia, E.rutaecarpa*	340.26169	65.1159 3	$C_{23}H_{33}NO$	$[M+H]^+$
Alkaloids	Quinolone alkaloids	dihydroevocarpine	*E.austrosinensis, E.fargesii, E.glabrifolia, E.rutaecarpa*	342.27048	54.3059 0	$C_{23}H_{35}NO$	$[M+H]^+$
Alkaloids	Quinolone alkaloids	1-methyl-2-[6-carbonyl-(*E*)-7-tridecenyl]-4(1*H*)-quinolone	*E.austrosinensis, E.glabrifolia*	354.22998	46.9021 0	$C_{23}H_{31}NO_2$	$[M+H]^+$

Class I	Class II	Compounds	Species	Molecular Weight	Rt (min)	Formula	Ionization model
Alkaloids	Quinolone alkaloids	evoprenine	*E.compacta, E.daniellii, E.delavayi, E.fargesii, E.fraxinifolia, E.glabrifolia, E.lepta, E.rutaecarpa, E.sutchuenensis*	355.18329	52.40749	$C_{21}H_{24}NO_4$	$[M+H]^+$
Alkaloids	Quinolone alkaloids	1-methyl-2-tetradecyl-4(1*H*)-quinolone	*E.compacta, E.daniellii, E.fargesii, E.fraxinifolia, E.glabrifolia, E.rutaecarpa*	356.11969	24.49990	$C_{24}H_{37}NO$	$[M+H]^+$
Alkaloids	Quinolone alkaloids	1-methyl-2-[13-hydroxyl-tridecenyl]-4(1*H*)-quinolone	*E.daniellii, E.fargesii, E.glabrifolia, E.rutaecarpa*	358.24548	29.49856	$C_{23}H_{35}NO_2$	$[M+H]^+$
Alkaloids	Quinolone alkaloids	1-methyl-2-[(*Z*)-6-pentadecenyl]-4(1*H*)-quinolone	*E.daniellii, E.glabrifolia, E.rutaecarpa*	366.22809	49.76320	$C_{25}H_{37}NO$	$[M+H]^+$
Alkaloids	Quinolone alkaloids	1-methyl-2-[(6*Z*,9*Z*)-6,9-pentadecadienyl]-4(1*H*)-quinolone	*E.compacta, E.fargesii, E.fraxinifolia, E.glabrifolia, E.rutaecarpa, E.sutchuenensis*	366.27820	53.86510	$C_{25}H_{35}NO$	$[M+H]^+$
Alkaloids	Quinolone alkaloids	2-nonyl-4(1*H*)-quinolone	*E.fraxinifolia, E.glabrifolia, E.lepta, E.rutaecarpa*	272.20270	55.34388	$C_{18}H_{25}NO$	$[M+H]^+$
Alkaloids	Quinolone alkaloids	(-) edulinine	*E.glabrifolia*	292.18768	47.41539	$C_{16}H_{21}NO_4$	$[M+H]^+$
Alkaloids	Quinolone alkaloids	2-tridecyl-4(1*H*)-quinolone	*E.compacta, E.delavayi, E.fargesii, E.fraxinifolia*	318.17288	68.95125	$C_{22}H_{23}NO$	$[M+H]^+$
Alkaloids	Quinolone alkaloids	1-methyl-2-[(4*Z*,7*Z*)-4,7-tridecadienyl]-4(1*H*)-quinolone	*E.compacta, E.fargesii, E.fraxinifolia, E.glabrifolia*	338.25650	58.39086	$C_{23}H_{31}NO$	$[M+H]^+$
Alkaloids	Quinolone alkaloids	1-methyl-2-[(*Z*)-10-pentadecenyl]-4(1*H*)-	*E.fargesii*	338.34430	76.40609	$C_{25}H_{37}NO$	$[M+H]^+$

Class I	Class II	Compounds	Species	Molecular Weight	Rt (min)	Formula	Ionization model
		quinolone					
Alkaloids	Quinolone alkaloids	1-methyl-2-[7-carbonyl-(*E*)-9-tridecenyl]-4(1*H*)-quinolone	*E.compacta, E.fargesii, E.glabrifolia, E.rutaecarpa*	354.22998	46.8401 3	$C_{23}H_{31}NO_2$	$[M+H]^+$
Alkaloids	Quinolone alkaloids	euocarpine D	*E.fargesii*	354.22998	59.6542 2	$C_{23}H_{31}NO_2$	$[M+H]^+$
Alkaloids	Quinolone alkaloids	1-methyl-2-pentadecenyl-4(1*H*)-quinolone	*E.compacta, E.fargesii, E.fraxinifolia, E.glabrifolia*	370.27798	70.1014 3	$C_{25}H_{39}NO$	$[M+H]^+$
Alkaloids	Quinolone alkaloids	1-methyl-2-[15-hydroxyl-pentadecenyl]-4(1*H*)-quinolone	*E.compacta, E.fargesii*	386.28729	66.6798 5	$C_{25}H_{39}NO_2$	$[M+H]^+$
Alkaloids	Quinolone alkaloids	1-methyl-2-octyl-4(1*H*)-quinolone	*E.ailanthifolia, E.compacta, E.glabrifolia*	272.20270	55.2916 5	$C_{18}H_{25}NO$	$[M+H]^+$
Alkaloids	Acridone alkaloids	xanthoxoline	*E.ailanthifolia, E.rutaecarpa*	272.06088	19.9766 6	$C_{15}H_{13}NO_4$	$[M+H]^+$
Alkaloids	Acridone alkaloids	evoxanthine	*E.sutchuenensis*	284.10715	4.27653	$C_{16}H_{13}NO_4$	$[M+H]^+$
Alkaloids	Acridone alkaloids	4-hydroxy-2,3-dimethoxy-10-methylacridone	*E.ailanthifolia*	302.13019	21.8966 2	$C_{16}H_{15}NO_5$	$[M+H]^+$
Alkaloids	Acridone alkaloids	melicopidine	*E.delavayi, E.fargesii, E.glabrifolia*	314.08099	0.76270	$C_{17}H_{15}NO_5$	$[M+H]^+$
Alkaloids	Acridone alkaloids	skimmianine	*E.compacta, E.fargesii, E.fraxinifolia, E.glabrifolia*	260.09630	9.43573	$C_{14}H_{13}NO_4$	$[M+H]^+$
Alkaloids	Acridone	kokusaginine	*E.sutchuenensis*	260.09839	10.8335	$C_{14}H_{13}NO_4$	$[M+H]^+$

Class I	Class II	Compounds	Species	Molecular Weight	Rt (min)	Formula	Ionization model
	alkaloids				8		
Alkaloids	Acridone alkaloids	isobalfourodine	*E.compacta*	290.13449	91.6419 9	$C_{16}H_{19}NO_4$	$[M+H]^+$
Alkaloids	Acridone alkaloids	2,3,4-trimethoxy-10-methylacridone	*E.daniellii*	300.12140	39.4928 9	$C_{17}H_{17}NO_4$	$[M+H]^+$
Alkaloids	Berberine alkaloids	coptisine	*E.austrosinensis, E.compacta, E.fargesii, E.glabrifolia, E.rutaecarpa, E.sutchuenensis*	321.10950	3.38678	$C_{19}H_{14}NO_4$	$[M+H]^+$
Alkaloids	Berberine alkaloids	epiberberine	*E.ailanthifolia, E.compacta, E.daniellii, E.delavayi, E.lepta*	337.09830	1.47941	$C_{20}H_{18}NO_4$	$[M+H]^+$
Alkaloids	Berberine alkaloids	berberine	*E.ailanthifolia, E.compacta, E.fargesii, E.rutaecarpa*	337.08078	1.33046	$C_{20}H_{18}NO_4$	$[M+H]^+$
Alkaloids	Berberine alkaloids	berberastine	*E.ailanthifolia, E.austrosinensis, E.compacta, E.delavayi, E.lepta, E.sutchuenensis*	353.13449	30.7228 2	$C_{20}H_{18}NO_5$	$[M+H]^+$
Alkaloids	Berberine alkaloids	jatrorrhizine	*E.ailanthifolia, E.daniellii, E.rutaecarpa*	339.12650	21.7421 8	$C_{20}H_{20}NO_4$	$[M+H]^+$
Alkaloids	Carboline alkaloids	2-methyl-6-hydroxy-1,2,3,4-tetrahydro-*β*-carboline	*E.daniellii, E.fargesii*	203.10730	22.6888 2	$C_{12}H_{14}N_2O$	$[M+H]^+$
Alkaloids	Carboline alkaloids	6-methoxy-*N*-methyl-1,2,3,4-tetrahydro-*β*-carboline	*E.ailanthifolia, E.daniellii, E.glabrifolia*	217.17690	47.7275 7	$C_{13}H_{16}N_2O$	$[M+H]^+$

Class I	Class II	Compounds	Species	Molecular Weight	Rt (min)	Formula	Ionization model
Alkaloids	Furanquinoline alkaloids	6-methoxydictamnine	*E.austrosinensis, E.lepta, E.rutaecarpa, E.sutchuenensis*	230.09249	3.62308	$C_{13}H_{11}NO_3$	$[M+H]^+$
Alkaloids	Furanquinoline alkaloids	evolitrine	*E.rutaecarpa*	230.14539	19.63435	$C_{13}H_{11}NO_3$	$[M+H]^+$
Alkaloids	Furanquinoline alkaloids	leptanoine A	*E.fargesii, E.rutaecarpa*	282.11649	0.95531	$C_{17}H_{15}NO_3$	$[M+H]^+$
Alkaloids	Furanquinoline alkaloids	melineurine	*E.compacta, E.fraxinifolia, E.glabrifolia, E.lepta, E.rutaecarpa, E.sutchuenensis*	284.11520	0.88546	$C_{17}H_{17}NO_3$	$[M+H]^+$
Alkaloids	Furanquinoline alkaloids	roxiamine C	*E.ailanthifolia, E.delavayi, E.rutaecarpa*	288.10788	24.06148	$C_{16}H_{17}NO_4$	$[M+H]^+$
Alkaloids	Furanquinoline alkaloids	buchapine	*E.rutaecarpa*	298.11011	1.10965	$C_{19}H_{23}NO_2$	$[M+H]^+$
Alkaloids	Furanquinoline alkaloids	leptanoine B	*E.glabrifolia, E.rutaecarpa, E.sutchuenensis*	312.12088	19.88019	$C_{18}H_{17}NO_4$	$[M+H]^+$
Alkaloids	Furanquinoline alkaloids	leptanoine C	*E.compacta, E.fraxinifolia, E.rutaecarpa, E.sutchuenensis*	314.14868	0.76055	$C_{18}H_{19}NO_4$	$[M+H]^+$
Alkaloids	Furanquinoline alkaloids	anhydroevoxine	*E.compacta, E.daniellii, E.fargesii, E.rutaecarpa, E.sutchuenensis*	330.12158	21.19140	$C_{18}H_{19}NO_5$	$[M+H]^+$
Alkaloids	Furanquinoline alkaloids	roxiamine B	*E.austrosinensis, E.compacta, E.delavayi*	328.12918	1.25079	$C_{18}H_{17}NO_5$	$[M+H]^+$
Alkaloids	Furanquinoline alkaloids	evolatine	*E.fargesii*	348.14268	1.54506	$C_{18}H_{21}NO_6$	$[M+H]^+$
Alkaloids	Furanquinoline alkaloids	evoxine	*E.lepta*	348.14270	2.38072	$C_{18}H_{21}NO_6$	$[M+H]^+$

Class I	Class II	Compounds	Species	Molecular Weight	Rt (min)	Formula	Ionization model
Alkaloids	Pyranquinoline alkaloids	flindersine	*E.ailanthifolia, E.austrosinensis, E.daniellii, E.glabrifolia, E.rutaecarpa, E.sutchuenensis*	228.09779	7.65223	$C_{14}H_{13}NO_2$	$[M+H]^+$
Alkaloids	Pyranquinoline alkaloids	haplamine	*E.austrosinensis, E.compacta, E.daniellii, E.fargesii, E.glabrifolia, E.lepta, E.rutaecarpa, E.sutchuenensis*	258.10590	14.8617 8	$C_{15}H_{15}NO_3$	$[M+H]^+$
Alkaloids	Pyranquinoline alkaloids	(-) ribalinine	*E.austrosinensis, E.glabrifolia, E.lepta, E.rutaecarpa, E.sutchuenensis*	260.11380	1.04642	$C_{15}H_{17}NO_3$	$[M+H]^+$
Alkaloids	Pyranquinoline alkaloids	(+)-ribahnine	*E.lepta, E.sutchuenensis*	260.11380	0.66055	$C_{15}H_{17}NO_4$	$[M+H]^+$
Alkaloids	Quinazoline alkaloids	vasicinone	*E.glabrifolia, E.rutaecarpa, E.sutchuenensis*	203.05280	0.87924	$C_{11}H_{10}N_2O_2$	$[M+H]^+$
Alkaloids	Quinazoline alkaloids	fumiquinazolines J	*E.austrosinensis, E.compacta, E.daniellii, E.delavayi, E.fargesii, E.glabrifolia, E.lepta, E.rutaecarpa*	357.14771	14.1150 0	$C_{21}H_{16}N_4O_2$	$[M+H]^+$
Alkaloids	Quinazoline alkaloids	fumiquinazolines A	*E.sutchuenensis*	446.17709	1.15185	$C_{24}H_{23}N_5O_4$	$[M+H]^+$
Alkaloids	Quinazoline alkaloids	fumiquinazolines E	*E.ailanthifolia, E.compacta, E.sutchuenensis*	476.19650	5.21834	$C_{25}H_{25}N_5O_5$	$[M+H]^+$
Alkaloids	Tryptamine alkaloids	fargesine	*E.austrosinensis, E.compacta, E.daniellii, E.delavayi, E.fargesii, E.fraxinifolia, E.glabrifolia, E.lepta, E.rutaecarpa*	219.10210	6.45477	$C_{12}H_{14}N_2O_2$	$[M+H]^+$
Alkaloids	Tryptamine alkaloids	*N*,*N*-dimethy-5-methoxytryptamine	*E.ailanthifolia, E.daniellii, E.rutaecarpa*	219.14450	1.60360	$C_{13}H_{18}N_2O$	$[M+H]^+$

Class I	Class II	Compounds	Species	Molecular Weight	Rt (min)	Formula	Ionization model
Alkaloids	Tryptamine alkaloids	5-methoxy-*N*,*N*-dimethyltryptamine-*Nβ*-oxide	*E.ailanthifolia, E.austrosinensis, E.compacta, E.fargesii, E.glabrifolia, E.lepta, E.rutaecarpa*	235.08780	19.97666	$C_{13}H_{18}N_2O_2$	$[M+H]^+$
Alkaloids	Tryptamine alkaloids	5-methoxy-*N*,*N*-dimethyltryptamine	*E.daniellii*	219.10210	8.07471	$C_{13}H_{18}N_2O$	$[M+H]^+$
Alkaloids	Tryptamine alkaloids	bufotenine 5-*O*-*β*-D-glucopyranoside *N*12-oxide	*E.compacta, E.fraxinifolia, E.glabrifolia*	384.18799	65.94263	$C_{18}H_{27}N_2O_7$	$[M+H]^+$
Alkaloids	Tryptamine alkaloids	bufotenine *N*12-oxide	*E.compacta, E.lepta*	222.10359	1.06986	$C_{12}H_{17}N_2O_2$	$[M+H]^+$
Alkaloids	Tryptamine alkaloids	plectocomine 12-methyl-5-*O*-*β*-D-glucopyranoside *N*12-oxide	*E.austrosinensis, E.lepta, E.rutaecarpa*	383.17139	5.09379	$C_{18}H_{25}N_2O_7$	$[M+H]^+$
Alkaloids	Tryptamine alkaloids	bufotenine	*E.austrosinensis*	205.08549	57.15387	$C_{12}H_{16}N_2O$	$[M+H]^+$
Alkaloids	Others	harmine	*E.glabrifolia, E.rutaecarpa*	213.17769	0.47736	$C_{13}H_{12}N_2O$	$[M+H]^+$
Alkaloids	Others	canthin-6-one	*E.ailanthifolia, E.austrosinensis, E.daniellii, E.rutaecarpa*	221.12810	1.13202	$C_{14}H_8N_2O$	$[M+H]^+$
Alkaloids	Others	higenamine	*E.ailanthifolia, E.austrosinensis, E.compacta, E.daniellii, E.rutaecarpa, E.sutchuenensis*	272.12390	1.30209	$C_{16}H_{17}NO_3$	$[M+H]^+$
Alkaloids	Others	ethylleptol B	*E.ailanthifolia, E.compacta, E.daniellii, E.delavayi, E.fargesii, E.glabrifolia, E.rutaecarpa*	293.20309	24.70381	$C_{17}H_{24}O_4$	$[M+H]^+$
Alkaloids	Others	shewanellines C	*E.compacta, E.rutaecarpa*	296.12663	1.04826	$C_{16}H_{13}N_3O_3$	$[M+H]^+$

Class I	Class II	Compounds	Species	Molecular Weight	Rt (min)	Formula	Ionization model
Alkaloids	Others	evellerine	*E.compacta, E.lepta*	318.12198	3.86699	$C_{17}H_{19}NO_5$	$[M+H]^+$
Alkaloids	Others	halfordinine	*E.ailanthifolia*	290.09958	24.4471 2	$C_{15}H_{15}NO_5$	$[M+H]^+$
Phenolic acids	Coumarins	xanthotoxin	*E.ailanthifolia, E.austrosinensis, E.delavayi, E.glabrifolia, E.lepta, E.rutaecarpa*	217.08549	1.19454	$C_{12}H_8O_4$	$[M+H]^+$
Phenolic acids	Coumarins	isopimpinellin	*E.compacta, E.daniellii, E.delavayi, E.fargesii, E.glabrifolia, E.rutaecarpa*	247.09720	2.19990	$C_{13}H_{10}O_5$	$[M+H]^+$
Phenolic acids	Coumarins	bergapten	*E.austrosinensis, E.delavayi*	217.04329	1.72412	$C_{12}H_8O_4$	$[M+H]^+$
Phenolic acids	Flavonoids	phellamurin	*E.austrosinensis, E.fargesii, E.glabrifolia*	519.35168	74.7508 9	$C_{26}H_{30}O_{11}$	$[M+H]^+$
Phenolic acids	Flavonoids	hesperidin	*E.compacta, E.glabrifolia*	611.15851	13.7558 3	$C_{28}H_{34}O_{15}$	$[M+H]^+$
Phenolic acids	Flavonoids	flavaprin	*E.compacta*	503.20078	13.7168 8	$C_{26}H_{30}O_{10}$	$[M+H]^+$
Phenolic acids	others	leptene B	*E.ailanthifolia, E.austrosinensis, E.compacta, E.daniellii, E.delavayi, E.fargesii, E.fraxinifolia, E.lepta, E.rutaecarpa*	247.11229	6.93464	$C_{15}H_{18}O_3$	$[M+H]^+$
Phenolic acids	others	isoevodionol	*E.ailanthifolia, E.austrosinensis, E.compacta, E.fargesii, E.fraxinifolia, E.glabrifolia, E.lepta, E.rutaecarpa*	249.11279	0.95264	$C_{14}H_{16}O_4$	$[M+H]^+$

Class I	Class II	Compounds	Species	Molecular Weight	Rt (min)	Formula	Ionization model
Phenolic acids	others	leptol B	*E.ailanthifolia, E.compacta, E.daniellii, E.delavayi, E.fargesii, E.glabrifolia, E.rutaecarpa*	265.11230	1.80185	$C_{15}H_{20}O_4$	$[M+H]^+$
Phenolic acids	others	emodin	*E.austrosinensis, E.rutaecarpa*	271.07735	22.80786	$C_{15}H_{10}O_5$	$[M+H]^+$
Phenolic acids	others	leptene A	*E.ailanthifolia, E.daniellii, E.fraxinifolia, E.lepta, E.rutaecarpa, E.sutchuenensis*	277.15640	22.08363	$C_{16}H_{20}O_4$	$[M+H]^+$
Phenolic acids	others	methylleptol B	*E.ailanthifolia, E.austrosinensis, E.compacta, E.daniellii, E.delavayi, E.fargesii, E.fraxinifolia, E.glabrifolia, E.lepta, E.sutchuenensis*	279.15100	27.55854	$C_{16}H_{22}O_4$	$[M+H]^+$
Phenolic acids	others	leptin A	*E.ailanthifolia, E.compacta, E.daniellii, E.fargesii, E.lepta, E.rutaecarpa, E.sutchuenensis*	283.10199	6.07034	$C_{14}H_{18}O_6$	$[M+H]^+$
Phenolic acids	others	leptin B	*E.compacta, E.rutaecarpa*	283.10199	4.18155	$C_{14}H_{18}O_6$	$[M+H]^+$
Phenolic acids	others	physcion	*E.ailanthifolia, E.austrosinensis, E.compacta, E.daniellii, E.fraxinifolia*	285.09790	37.56786	$C_{16}H_{12}O_5$	$[M+H]^+$
Phenolic acids	others	alloevodione	*E.rutaecarpa*	293.20309	27.58928	$C_{16}H_{20}O_5$	$[M+H]^+$
Phenolic acids	others	leptol A	*E.glabrifolia*	295.11688	1.12054	$C_{16}H_{22}O_5$	$[M+H]^+$
Phenolic	others	2-(1’-geranyloxy)-4,6-	*E.compacta, E.daniellii, E.lepta*	305.10941	23.1827	$C_{18}H_{24}O_4$	$[M+H]^+$

Class I	Class II	Compounds	Species	Molecular Weight	Rt (min)	Formula	Ionization model
acids		dihydroxyacetophenone			0		
Phenolic acids	others	leptin C	*E.ailanthifolia, E.austrosinensis, E.compacta, E.daniellii, E.delavayi, E.fargesii, E.fraxinifolia, E.glabrifolia, E.lepta, E.rutaecarpa, E.sutchuenensis*	311.14319	5.67042	$C_{16}H_{22}O_6$	$[M+H]^+$
Phenolic acids	others	leucomidines C	*E.austrosinensis, E.compacta, E.daniellii, E.fargesii, E.fraxinifolia, E.glabrifolia, E.rutaecarpa*	315.19901	38.58466	$C_{18}H_{22}N_2O_3$	$[M+H]^+$
Phenolic acids	others	melofolione Ⅰa	*E.austrosinensis, E.compacta, E.glabrifolia, E.rutaecarpa*	319.11050	1.26927	$C_{18}H_{22}O_5$	$[M+H]^+$
Phenolic acids	others	leucomidines A	*E.ailanthifolia, E.fargesii, E.lepta, E.rutaecarpa, E.sutchuenensis*	325.13454	5.99697	$C_{19}H_{20}N_2O_3$	$[M+H]^+$
Phenolic acids	others	leucomidines B	*E.compacta, E.fargesii, E.glabrifolia, E.lepta, E.rutaecarpa*	341.16058	13.89834	$C_{20}H_{24}N_2O_3$	$[M+H]^+$
Phenolic acids	others	7,4-dihydroxy-3,5,3’-trimethoxyflavone	*E.compacta, E.fargesii, E.glabrifolia, E.lepta, E.rutaecarpa*	345.10529	26.24281	$C_{18}H_{16}O_7$	$[M+H]^+$
Phenolic acids	others	floridolide B		353.22089	14.98667	$C_{20}H_{32}O_5$	$[M+H]^+$
Phenolic acids	others	floridiolic acid	*E.austrosinensis, E.compacta, E.fargesii, E.fraxinifolia, E.glabrifolia, E.lepta, E.rutaecarpa, E.sutchuenensis*	353.22430	47.22457	$C_{21}H_{36}O_4$	$[M+H]^+$
Phenolic acids	others	fumiquinazolines F	*E.ailanthifolia, E.daniellii, E.fargesii, E.lepta, E.sutchuenensis*	359.15369	3.01148	$C_{21}H_{18}N_4O_2$	$[M+H]^+$

Class I	Class II	Compounds	Species	Molecular Weight	Rt (min)	Formula	Ionization model
Phenolic acids	others	floribundic acid	*E.compacta, E.delavayi, E.glabrifolia, E.rutaecarpa*	359.17178	4.64139	$C_{21}H_{26}O_5$	$[M+H]^+$
Phenolic acids	others	3-*O*-*β*-D- caffeoylquinic acid methyl ester	*E.compacta, E.daniellii, E.delavayi, E.fraxinifolia, E.rutaecarpa*	369.12128	1.20749	$C_{17}H_{20}O_9$	$[M+H]^+$
Phenolic acids	others	meliternatin	*E.rutaecarpa*	371.15530	2.17740	$C_{19}H_{14}O_8$	$[M+H]^+$
Phenolic acids	others	syringin	*E.ailanthifolia, E.compacta, E.fargesii, E.glabrifolia, E.lepta, E.rutaecarpa, E.sutchuenensis*	373.14468	14.9371 6	$C_{17}H_{24}O_9$	$[M+H]^+$
Phenolic acids	others	5,4'-dihydroxy-3,7,3'-trimethoxyflavone	*E.glabrifolia*	375.12659	1.71843	$C_{19}H_{18}O_8$	$[M+H]^+$
Phenolic acids	others	isobauerenol	*E.ailanthifolia, E.compacta, E.fargesii, E.glabrifolia, E.rutaecarpa*	385.27438	62.6268 8	$C_{27}H_{44}O$	$[M+H]^+$
Phenolic acids	others	citroside A	*E.fargesii, E.rutaecarpa, E.sutchuenensis*	387.15704	5.18553	$C_{19}H_{30}O_8$	$[M+H]^+$
Phenolic acids	others	austroside B	*E.ailanthifolia, E.compacta, E.fargesii, E.fraxinifolia, E.glabrifolia, E.lepta, E.rutaecarpa, E.sutchuenensis*	387.16989	2.20911	$C_{19}H_{30}O_8$	$[M+H]^+$
Phenolic acids	others	corchoionoside B	*E.ailanthifolia, E.compacta, E.fargesii, E.fraxinifolia, E.rutaecarpa, E.sutchuenensis*	401.14209	2.37908	$C_{19}H_{28}O_9$	$[M+H]^+$
Phenolic acids	others	austroside A	*E.ailanthifolia, E.compacta, E.fargesii, E.fraxinifolia, E.glabrifolia, E.rutaecarpa*	403.13895	6.27573	$C_{19}H_{30}O_9$	$[M+H]^+$
Phenolic acids	others	isolimonexic acid	*E.austrosinensis, E.rutaecarpa, E.sutchuenensis*	503.20078	10.2755 8	$C_{26}H_{30}O_{10}$	$[M+H]^+$

Class I	Class II	Compounds	Species	Molecular Weight	Rt (min)	Formula	Ionization model
Phenolic acids	others	epimedoside C	*E.ailanthifolia, E.compacta, E.daniellii, E.delavayi, E.lepta*	517.22107	31.91394	$C_{26}H_{28}O_{11}$	$[M+H]^+$
Phenolic acids	others	ruteavine acetate	*E.austrosinensis, E.compacta, E.delavayi, E.fargesii, E.fraxinifolia, E.glabrifolia, E.rutaecarpa*	529.32281	48.28838	$C_{28}H_{32}O_{10}$	$[M+H]^+$
Phenolic acids	others	dichromene A	*E.compacta, E.fargesii, E.fraxinifolia, E.glabrifolia, E.rutaecarpa*	571.18207	20.58700	$C_{32}H_{42}O_9$	$[M+H]^+$
Phenolic acids	others	dichromene B	*E.rutaecarpa*	571.43311	79.31596	$C_{32}H_{42}O_9$	$[M+H]^+$
Phenolic acids	others	shewanellines A	*E.lepta, E.sutchuenensis*	260.09839	0.92638	$C_{17}H_{13}N_2O_2$	$[M+H]^+$
Phenolic acids	others	leptonol methylleptol	*E.austrosinensis, E.compacta, E.fargesii, E.glabrifolia, E.rutaecarpa*	279.13120	27.79548	$C_{15}H_{18}O_5$	$[M+H]^+$
Phenolic acids	others	quercetin	*E.compacta, E.delavayi, E.fargesii, E.glabrifolia*	303.04379	13.80805	$C_{15}H_{10}O_7$	$[M+H]^+$
Phenolic acids	others	2-(1'-geranyloxy)-4,6,*β*-trihydroxyacetophenone	*E.ailanthifolia, E.austrosinensis, E.glabrifolia, E.rutaecarpa, E.sutchuenensis*	305.22549	73.30245	$C_{18}H_{24}O_5$	$[M+H]^+$
Phenolic acids	others	ethylleptol A	*E.glabrifolia*	323.18338	71.04980	$C_{18}H_{26}O_5$	$[M+H]^+$
Phenolic acids	others	floridolide A	*E.austrosinensis, E.compacta, E.fargesii, E.fraxinifolia, E.glabrifolia*	337.23859	56.88998	$C_{20}H_{32}O_4$	$[M+H]^+$
Phenolic acids	others	hydroxy lacton	*E.ailanthifolia, E.austrosinensis, E.compacta, E.fargesii, E.glabrifolia, E.lepta*	363.16440	72.45423	$C_{20}H_{26}O_6$	$[M+H]^+$
Phenolic	others	ternatin	*E.compacta, E.fargesii, E.rutaecarpa*	375.09885	1.39462	$C_{19}H_{18}O_8$	$[M+H]^+$

Class I	Class II	Compounds	Species	Molecular Weight	Rt (min)	Formula	Ionization model
acids							
Phenolic acids	others	dihydrophaseic acid 4'-*O*-*β*-D-glucopyranoside	*E.ailanthifolia, E.compacta, E.delavayi, E.glabrifolia, E.sutchuenensis*	384.18799	65.94263	$C_{21}H_{32}O_{10}$	$[M+H]^+$
Phenolic acids	others	methylleptol A	*E.compacta, E.daniellii, E.delavayi, E.fargesii, E.glabrifolia, E.rutaecarpa*	309.15390	3.57367	$C_{17}H_{24}O_5$	$[M+H]^+$
Phenolic acids	others	3-methylquercetin(5,4'-dihydroxy-3,7,8,3'-tetramethoxyflavone)	*E.compacta, E.glabrifolia, E.sutchuenensis*	317.07518	20.63562	$C_{16}H_{12}O_7$	$[M+H]^+$
Phenolic acids	others	melofolione Ⅰ b	*E.ailanthifolia, E.austrosinensis, E.compacta, E.daniellii, E.delavayi, E.fargesii, E.fraxinifolia, E.glabrifolia, E.lepta, E.rutaecarpa, E.sutchuenensis*	319.14468	16.07384	$C_{18}H_{22}O_5$	$[M+H]^+$
Phenolic acids	others	4-(1'-geranyloxy)-*β*,2,6-trihydroxyacetophenone	*E.lepta*	321.16071	28.73671	$C_{18}H_{24}O_5$	$[M+H]^+$
Phenolic acids	others	4-(1'-geranyloxy)-*β*,2,6-dihydroxyacetophenone	*E.compacta, E.lepta, E.rutaecarpa*	389.22079	55.77020	$C_{18}H_{24}O_4$	$[M+H]^+$
Phenolic acids	others	4-(1'-geranyloxy)-2,6,*β*-trihydroxy-3-dimethylallylacetophenone	*E.ailanthifolia, E.austrosinensis, E.daniellii, E.delavayi, E.fargesii, E.fraxinifolia, E.lepta, E.rutaecarpa*	389.23239	32.30911	$C_{23}H_{32}O_5$	$[M+H]^+$
Phenolic acids	others	chrysophanol	*E.ailanthifolia, E.compacta*	255.08670	35.66378	$C_{15}H_{10}O_4$	$[M+H]^+$
Phenolic acids	others	3,7-dimethylkaempferol	*E.compacta, E.fargesii*	345.10529	1.06125	$C_{18}H_{16}O_7$	$[M+H]^+$
Phenolic	others	diosmetin-7-*O*-*β*-D-	*E.ailanthifolia*	463.23499	20.3475	$C_{22}H_{22}O_{11}$	$[M+H]^+$

Class I	Class II	Compounds	Species	Molecular Weight	Rt (min)	Formula	Ionization model
acids		glucopyranoside			0		
Terpene	limonins	limonin	*E.ailanthifolia, E.austrosinensis, E.compacta, E.daniellii, E.fargesii, E.fraxinifolia, E.glabrifolia, E.rutaecarpa, E.sutchuenensis*	471.19189	16.4031 9	$C_{26}H_{30}O_8$	$[M+H]^+$
Terpene	limonins	evodol	*E.austrosinensis, E.compacta, E.daniellii, E.delavayi, E.fargesii, E.fraxinifolia, E.glabrifolia, E.lepta, E.rutaecarpa, E.sutchuenensis*	485.32550	63.2982 9	$C_{26}H_{28}O_9$	$[M+H]^+$
Terpene	limonins	rutaevine	*E.austrosinensis, E.delavayi, E.fargesii, E.fraxinifolia, E.glabrifolia, E.rutaecarpa, E.sutchuenensis*	487.36829	77.0922 1	$C_{26}H_{30}O_9$	$[M+H]^+$
Terpene	limonins	shihulimonin A1	*E.austrosinensis, E.compacta, E.delavayi, E.fargesii, E.fraxinifolia, E.glabrifolia, E.rutaecarpa*	503.20078	18.5426 2	$C_{26}H_{30}O_{10}$	$[M+H]^+$
Terpene	limonins	euodirutaecins B	*E.fargesii, E.glabrifolia*	517.35150	75.2703 8	$C_{26}H_{28}O_{11}$	$[M+H]^+$
Terpene	limonins	euodirutaecins A	*E.austrosinensis, E.fargesii, E.fraxinifolia, E.glabrifolia*	517.37317	66.3468 1	$C_{26}H_{28}O_{11}$	$[M+H]^+$
Alkaloids	Indole quinazoline alkaloids	13-methyl-13H-indolo [2', 3':3, 4]pyrido[2,1,*β*]quinazolin-5-one	*E.austrosinensis, E.fargesii, E.glabrifolia*	300.35172	49.6538 4	$C_{19}H_{13}N_3O$	$[M+H]^+$
Alkaloids	Indole	13*β*-acetongl-hydroxy-	*E.fargesii, E.rutaecarpa*	360.44376	24.3485	$C_{22}H_{21}N_3O_2$	$[M+H]^+$

Class I	Class II	Compounds	Species	Molecular Weight	Rt (min)	Formula	Ionization model
	quinazoline alkaloids	evodiamine			9		
Alkaloids	Indole quinazoline alkaloids	7*β*-hydroxyrutaecarpine	*E.glabrifolia*	304.32721	18.3454 1	$C_{18}H_{13}N_3O_2$	$[M+H]^+$
Phenolic acids	Flavonoids	sinapyl alcohol 9-*O*-feruloyl-4-*O*-*β*-D-glucopyanoside	*E.glabrifolia*	549.56239	29.6759 4	$C_{27}H_{32}O_{12}$	$[M+H]^+$
