# Supplementary material for: Comprehensive Analysis of 11 Species of Euodia (Rutaceae) by Untargeted LC-IT-TOF/MS Metabolomics and In Vitro Functional Methods
Source: Molecules. 2024 Feb 28;29(5):1059. doi: 10.3390/molecules29051059 (PMC10934029; doi:10.3390/molecules29051059)
Supplement: Supplementary file 1 [file molecules-29-01059-s001.zip › Supplementary file 4.pdf]

Table S4. Plant material information supplement

No.	Species	Plant material pictures	Identification characteristics of plant materials
1	*Euodia lepta (E. lepta)*	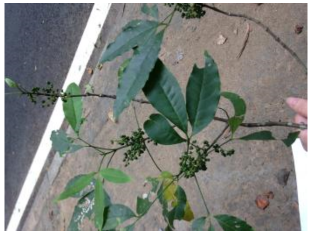	Shrubs or trees, rarely scandent, 1-14 m tall, dioecious or rarely monoclinous; Leaves 3-foliolate (occasional leaves 1-foliolate), leaflet blades ovate-elliptic, elliptic, elliptic-obovate, or narrowly so, in terminal leaflet 6-22 × 2-8 cm, glabrous or puberulent on midvein especially abaxially, apex acuminate or subcaudate; Fruit follicles subglobose to ellipsoid to obovoid, (3-)3.5-7.5 mm, glabrous or sparsely puberulent, Seeds subglobose to ovoid to ellipsoid.
2	*Euodia rutaecarpa (E. rutaecarpa)*	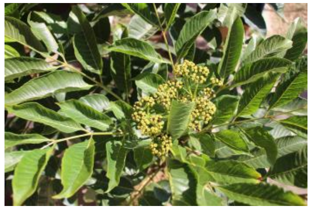	Shrubs or trees, to 9 m tall. Leaves 15-40 cm, (3 or)5-13(or 15)-foliolate; Leaflet blades elliptic to ovate or sometimes lanceolate, oblanceolate, or obovate, 4.5-17 × 2-8 cm, abaxially rarely slightly glaucous and not papillate; Fruit usually 5-carpelled; follicles subglobose, 3.5-6 mm, glabrous or sometimes with sparse trichomes, apex not beaked.
3	*Euodia compacta (E. compacta)*[*]	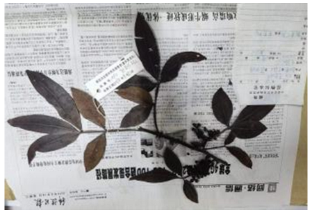	Shrubs or trees, about 3 m tall. Branches dark purplish red, glabrous or barely glabrous. Leaflet dry dark reddish-brown, often slightly wrinkled; Leaves have leaflets 5-9, papery, entire margin, ovate-elliptic or lanceolate, usually ovate located lower on leaf axis, 6-16 cm long and 2-6 cm wide, apex long acuminate, base broadly cuneate; Fruit sequence usually less than 8 cm long, fruits densely clustered, bright red or purplish red, inner pericarp slightly thicker than outer pericarp, nearly woody after drying, brown, 1 seed per carpopole; The seeds are 4-5 mm long and 3.5-4.5 mm wide, blue-black and shiny.
4	*Euodia austrosinensis (E. austrosinensis)*	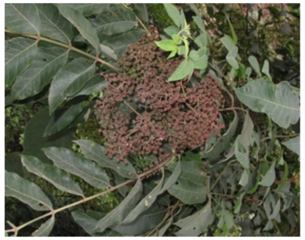	Trees to 20 m tall. Leaves 20-35 cm, 7-11-foliolate; leaflet blades broadly elliptic or sometimes ovate, lanceolate, obovate, or oblanceolate, 5.5-14.5(-17) × 2.5-7(-8.5) cm, abaxially glaucous and finely papillate, secondary veins 9-12 on each side of midvein, reticulate veinlets abaxially usually ± conspicuous and ± loose, base in lateral leaflets obtuse to nearly rounded and in terminal leaflet cuneate, margin entire, apex acuminate; Follicles trigonous to subtrigonous, ca. 3.5 mm, glabrous or rarely with a few scattered trichomes, apex not beaked; endocarp glabrous.

5	*Euodia glabrifolia* *(E. glabrifolia)*	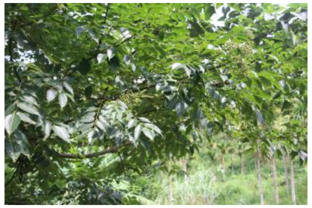	Shrubs or trees, to 20 m tall; Leaves 14-38 cm, (3 or)5-19-foliolate; leaflet blades broadly ovate to lanceolate or less often elliptic or elliptic-oblong, 4-15 × 1.7-6 cm, abaxially usually glaucous and not papillate, in terminal leaflet narrowly cuneate to cuneate, margin entire or ± crenulate, apex acuminate; Fruit usually 5-carpelled; follicles trigonous, 3.5-5 mm, laterally sparsely to densely appressed pubescent, otherwise glabrous, apex not beaked; endocarp sparsely to ± densely pubescent.
6	*Euodia ailanthifolia* *(E. ailanthifolia)*	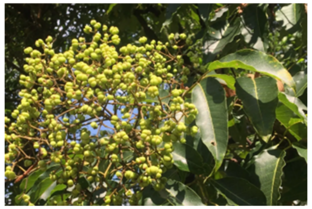	The trees are usually 8-15 m tall. The shoots are dark purple. Leaves have 7-13 leaflets, ovate-lanceolate or lanceolate, 6-12 cm long and 3-6 cm wide, often ovate at the base of the leaf axis, slanted on one side of the base at the upper part, with marked asymmetry on both sides; Dorsal leaf gray-green or pale gray, leaf margin with fine obtuse teeth or nearly entire margin; The stalk is not more than 5 mm long, the schizocarp is 4-5 mm long, the two sides are covered with short gray hairs, and each schizocarp has 1 seed.
7	*Euodia fargesii (E. fargesii)**	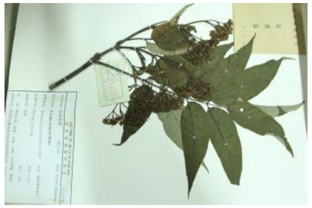	A 17 m tall tree with a diameter of 40 cm at breast height. Bark smooth, dark gray, shoots purple brown, scattered small skin holes; Leaves have 5-9 leaflets, rarely 11, obovate to oblique lanceolate, 8-16 cm long, 3-7 cm wide, smaller at the base of the leaf axis, usually round on one side, cuneapical on the other, very asymmetric on both sides, glabrous, dorsal gray-green; Mature carpellary 5-4, rare 3, purple red, dry after the color is darker, each carpellary has 1 seed.
8	*Euodia sutchuenensis* *(E. sutchuenensis)**	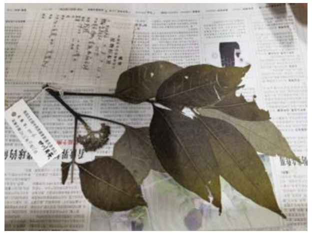	Tree, about 20 m tall, diameter 60 cm. Bark gray, not dehiscent, with small, oblate, slightly raised, yellow-gray pores, shoots and bud scales dark purplish red, densely covered with fine hair, pith large, inflorescence axis and branches densely covered with brown or red rust short hair; Leaves have 7-11 leaflets with lobed teeth, thickly papered, ovate-elliptic or oblong, 10-20 cm long and 5-9 cm wide; Schizocarpus purplish red, the back is sparsely hairy, the two sides are densely coated, 5-6 mm long, the tip has 1-2 mm long awn tip, the inner pericarp is osteoid, waxy yellow, the top is short pointed, the inner and outer pericarp are about equal thickness, each schizocarpus has 2 seeds.

9	*Euodia daniellii (E. daniellii)*	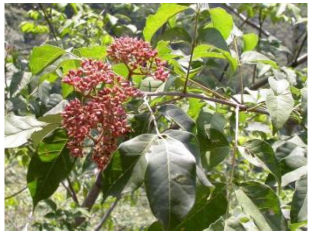	Shrubs or trees, to 20 m tall. Leaves 15-44 cm, 5-9 (or 11) -foliolate; leaflet blades broadly ovate to lanceolate or rarely elliptic or elliptic-oblong, 5-18.5 × 2.5-10.5 cm, abaxially sometimes ± glaucous and not papillate, secondary veins 7-14 on each side of midvein; reticulate veinlets abaxially usually inconspicuous, base in lateral leaflets narrowly cuneate, subtruncate, subrounded, or subcordate. Fruit usually 5-carpelled; follicles narrowly pyriform, 5-11 mm, sparsely to ± densely spreading pubescent, apex beaked; endocarp glabrous.
10	*Euodia delavayi (E. delavayi)*	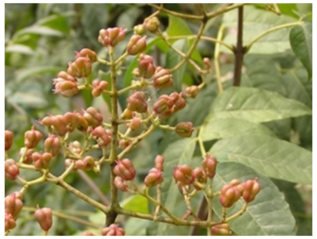	The tree is 10-20 meters tall. The leaves have 7-9 leaflets, oblong or oval, 8-15 cm long and 4-6 cm wide, apical or acuminate at the top, rounded or broadly cuneate at the base, symmetrical or sharply skewed on one side or unequal on both sides; Slightly thick-papery, leaf margin with very fine blunt teeth or close to the entire margin, the back of the leaf with tufts on the axillary of the vein, or along the sides of the midvein with long hairs; Schizocarp purplish red, glabrous back, short hairs on both sides, 6-7.5 mm long, tip with 2.5-3.5 mm long awns, inner peel waxy yellow.
11	*Euodia fraxinifolia (E. fraxinifolia)*	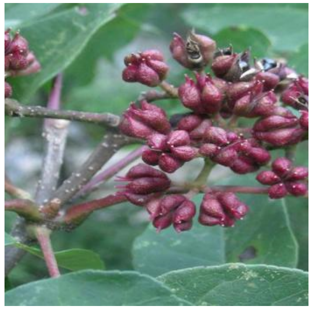	Trees to 12 m tall. Leaves 23-67 cm, 5-15-foliolate; leaflet blades lanceolate or occasionally ovate, elliptic-oblong, or elliptic, 9-25 × 3.5-8.5 cm, abaxially often glaucous and sometimes subpapillate, secondary veins 13-22 on each side of midvein, reticulate veinlets abaxially usually inconspicuous, base in lateral leaflets rounded to narrowly cuneate and in terminal leaflet cuneate, margin crenulate or rarely entire, apex acuminate; follicles compressed subglobose, 5.5-10 mm, glabrous to sparsely pubescent, apex not beaked; endocarp glabrous or sparsely pubescent. Seeds 2 per follicle, subtrigonous, 4-5 mm, attached in dehisced follicle to a fleshy funiculate aril; seed coat with thick inner layer of dense black sclerenchyma and spongy outer layer bounded externally by a reddish brown to brownish black shiny pellicle.

Table S5. Collection time Environmental status

Species	collection year	GPS coordinates of collection site	average annual precipitation	temperature	sunlight
Evodia lepta (E. lepta)	2017.10.08	21°55′27.26″N 101°15′27.44″E	1333.47	19.5	2500
Evodia lepta (E. lepta)	2017.10.11	22°50′7.36″N 100°59′56.94″E	1023.11	22.5	2049
Evodia lepta (E. lepta)	2017.10.17	24°53′49.81″N 110°57′51.90″E	1770	23	1816.8
Evodia lepta (E. lepta)	2016.08.01	18°42′17.72″N 109°51′17.81″E	3465	22.5	1282.1
Evodia lepta (E. lepta)	2016.08.01	18°39′20.93″N 109°54′29.38″E	3465	22.5	1282.1
Evodia lepta (E. lepta)	2016.08.01	18°40′28.84″N 109°54′10.84″E	3465	22.5	1282.1
Evodia rutaecarpa (E. rutaecarpa)	2018.09.24	32°03′09.68″N 118°50′03.20″E	1094	16.5	1960
Evodia rutaecarpa (E. rutaecarpa)	2016.09.15	26°16′12.57″N 106°58′49.82″E	1449.7	15.5	1342.6
Evodia rutaecarpa (E. rutaecarpa)	2016.08.27	27°36′20.64″N 105°50′46.37″E	1449.7	15.5	1342.6
Evodia rutaecarpa (E. rutaecarpa)	2016.08.31	25°54′0.55″N 104°59′43.88″E	1008	16	2098
Evodia rutaecarpa (E. rutaecarpa)	2018.09.21	27°50′46.12″N 109°14′29.63″E	1600	17	1303.1
Evodia rutaecarpa (E. rutaecarpa)	2018.09.22	27°13′16.53″N 107°56′22.79″E	1562	18.5	2237
Evodia compacta (E. compacta)	2018.08.21	30°32′45.75″N 114°25′18.06″E	1623.6	23	1608
Evodia compacta (E. compacta)	2018.09.10	30°15′10.26″N 120°07′05.53″E	1213.5	16.5	2017
Evodia compacta (E. compacta)	2018.09.10	30°15′13.40″N 120°07′02.75″E	1213.5	16.5	2017
Evodia austrosinensis (E. austrosinensis)	2018.10.08	21°55′27.26″N 101°15′27.44″E	1304.2	22	1504
Evodia austrosinensis (E. austrosinensis)	2018.10.17	24°53′54.01″N 110°59′19.65″E	1770	23	1816.8
Evodia austrosinensis (E. austrosinensis)	2018.08.30	21°55′27.26″N 101°15′27.44″E	1304.2	22	1504
Evodia glabrifolia (E. glabrifolia)	2016.08.01	18°41′16.85″N 109°43′44.21″E	3465	22.5	1282.1
Evodia glabrifolia (E. glabrifolia)	2016.08.01	18°40′51.09″N 109°51′35.11″E	3465	22.5	1282.1
Evodia glabrifolia (E. glabrifolia)	2016.08.01	18°42′31.58″N 109°50′28.71″E	3465	22.5	1282.1
Evodia ailanthifolia (E. ailanthifolia)	2018.10.08	21°55′27.26″N 101°15′27.44″E	1304.2	22	1504
Evodia ailanthifolia (E. ailanthifolia)	2018.10.11	22°50′7.36″N 100°59′56.94″E	1333.47	19.5	2500
Evodia ailanthifolia (E. ailanthifolia)	2018.08.29	22°46′21.55″N 100°59′31.66″E	1333.47	19.5	2500

Evodia fargesii (E. fargesii)	2016.09.10	30°14′57.93″N 120°07′06.80″E	1213.5	16.5	2017
Evodia fargesii (E. fargesii)	2016.08.31	25°47′13.18″N 104°58′17.48″E	1008	16	2098
Evodia fargesii (E. fargesii)	2016.09.18	28°06′11.01″N 113°01′55.86″E	1900	22	1850
Eevodia sutchuenensis (E. sutchuenensis)	2016.08.16	29°04′20.90″N 107°09′38.44″E	1076	17.5	1400
Eevodia sutchuenensis (E. sutchuenensis)	2016.08.17	29°05′59.06″N 107°10′06.24″E	1076	17.5	1400
Eevodia sutchuenensis (E. sutchuenensis)	2018.08.11	29°04′51.28″N 107°08′49.46″E	1076	17.5	1400
Evodia daniellii (E. daniellii)	2017.09.16	41°54′33.51″N 123°36′01.37″E			
Evodia daniellii (E. daniellii)	2017.09.20	34°42′49.50″N 119°22′41.94″E	660	12.5	2541.1
Evodia daniellii (E. daniellii)	2017.09.24	36°38′48.54″N 117°01′18.37″E	638.8	13.5	2367.2
Evodia daniellii (E. daniellii)	2017.09.24	36°38′46.09″N 117°01′21.31″E	638.8	13.5	2367.2
Evodia delavayi (E. delavayi)	2018.10.24	28°26′32.58″N 98°54′49.57″E	870.6	15.5	2088.8
Evodia delavayi (E. delavayi)	2018.08.04	26°52′10.29″N 100°13′54.36″E	1333.47	19.5	2500
Evodia delavayi (E. delavayi)	2018.08.10	29°03′59.43″N 107°08′43.13″E	1076	17.5	1400
Evodia fraxinifolia (E. fraxinifolia)	2018.08.08	24°45′51.16″N 100°30′10.87″E	870.6	15.5	2088.8
Evodia fraxinifolia (E. fraxinifolia)	2018.08.02	24°23′28.77″N 100°46′47.38″E	800	17	2323
Evodia fraxinifolia (E. fraxinifolia)	2018.08.06	25°27′45.18″N 98°45′54.14″E	1562	19	2237

Table S6. Summary of method validation.

**Parameter**	**Value**
Retention time stability (RSD, %)	0.10
Sample stability (RSD, %)	2.54
Intra-day (RSD, %)	1.09

Inter-day (RSD, %)	2.2<br>3
Repeatability (RSD, %)	3.9<br>8
